# A locally administered single-cycle influenza vaccine expressing a non-fusogenic stabilized hemagglutinin stimulates strong T-cell and neutralizing antibody immunity

**DOI:** 10.1128/jvi.00331-24

**Published:** 2025-01-27

**Authors:** Holly L. Sadler, Pramila Rijal, Tiong Kit Tan, Alain R. M. Townsend

**Affiliations:** 1 MRC Translational Immune Discovery Unit, Weatherall Institute of Molecular Medicine, University of Oxford 6396 , Oxford, United Kingdom; 2 BBSRC Doctoral Training Partnership, University of Oxford 6396 , Oxford, United Kingdom; 3 Centre for Translational Immunology, Chinese Academy of Medical Sciences Oxford Institute, Nuffield Department of Medicine, University of Oxford 105596 , Oxford, United Kingdom; Emory University School of Medicine, Atlanta, Georgia, USA

**Keywords:** influenza vaccines, T-cell immunity, neutralizing antibodies, hemagglutinin

## Abstract

Current influenza vaccination approaches protect against specific viral strains, but do not consistently induce broad and long-lasting protection to the diversity of circulating influenza viruses. Single-cycle viruses delivered to the respiratory tract may offer a promising solution as they safely express a diverse array of viral antigens by undergoing just one round of cell infection in their host and stimulate broadly protective resident memory T-cell responses in the lung. We have previously developed a vaccine candidate called S-FLU, which is limited to a single cycle of infection by inactivation of the hemagglutinin signal sequence and induces a broadly cross-reactive T-cell response and antibodies to neuraminidase, but fails to induce neutralizing antibodies to hemagglutinin after intranasal administration. This study describes the development of CLEARFLU, a derivative of S-FLU, which is designed to add a neutralizing antibody response to hemagglutinin. In contrast to S-FLU, which does not express a hemagglutinin molecule at the infected cell surface, CLEARFLU viruses express a stabilized non-fusogenic hemagglutinin. They are equally limited to a single cycle of infection, but induce a neutralizing antibody response to the expressed hemagglutinin in addition to the cytotoxic T-lymphocyte (CTL) responses to internal proteins and antibodies to neuraminidase induced by S-FLU. This represents a notable advantage as CLEARFLU viruses may provide sterile immunity against strain-matched challenge as well as non-sterile protection against a broad range of influenza viruses.

**IMPORTANCE:**

Influenza is a serious public health concern, causing seasonal epidemics as well as pandemics in people. Influenza can also cause severe agricultural losses due to its circulation in farmed poultry and swine. A major challenge in the control of influenza is the diversity of circulating viruses. Developing vaccines that stimulate immunity to a wide array of influenza viruses is therefore important for protecting human and animal populations from disease and death. In this study, we describe an approach for developing influenza vaccines that trigger immune mechanisms shown to induce broad protection against a diversity of viruses, while also conserving the strong protection against specific strains observed in existing vaccines.

## INTRODUCTION

Viruses that undergo a single replication cycle offer a promising platform for generating broadly protective immune responses to influenza (1–3). They are designed to lack a functional copy of one or more of the genes required for influenza replication and can only be propagated when the related gene product is supplied in *trans*. While single-cycle viruses can be propagated to high titer in permissive cell culture, they are able to undergo only a single cycle of cell infection in their natural hosts.

Single-cycle influenza viruses (SCIV) with a functioning polymerase undergo genome replication and expression in their host, leading to amplification of viral epitopes inside infected cells and cell surface expression of encoded viral coat proteins. As SCIV typically contain all but one intact viral segment, responses can be targeted to a wide array of RNA, peptide, and protein components. SCIV are therefore capable of generating strong antibody and T-cell responses, including broadly protective cytotoxic T-cell responses to peptides from highly conserved internal core proteins (4). SCIV hold an advantage over conventional live-attenuated vaccines in that they infect very few cells and so are unlikely to cause pathology or mutate or reassort into virulent forms. SCIV can be safely administered by aerosol to immunize the lungs, which is associated with local immunity through tissue-resident T cells (5–7) and mucosal IgA (8).

Multiplasmid transfection is commonly used to produce influenza viruses *de novo* in cell culture (9–11). To generate single-cycle viruses, one or more producer plasmids can be mutated or omitted. SCIV vaccine candidates with deficiencies in various genes have been developed, including in matrix 2 (3), neuraminidase, the polymerase genes, and hemagglutinin. Inactivating hemagglutinin is particularly advantageous as it prevents functional hemagglutinin reassortment. Hemagglutinin activity is also easy to compensate for *in trans* by growing viruses in cells, which express functional coating hemagglutinin on their surface. Altering the coat protein in the producer cell lines (pseudotyping) can allow single-cycle viruses with the same core genes but varying coating hemagglutinins to be produced quickly from a single-seed virus. This has the advantage that the coating hemagglutinin can be selected to avoid prior immunity and enhance the cell-mediated immune response (12).

We have previously described the single-cycle virus S-FLU, which is produced by inactivation of the hemagglutinin signal sequence, generating a viral RNA called S-HA (signal minus hemagglutinin) (13). Like for most other SCIVs lacking hemagglutinin activity, no hemagglutinin epitopes are presented on the surface of cells infected with S-FLU. In other designs, the majority of the hemagglutinin coding sequence can be replaced by other genes, for example, fluorescent proteins to follow cell infection (14), or NY-ESO-1 to induce tumor immunity (12). S-FLU viruses have been helpful laboratory tools for evaluating the susceptibility of different coat proteins to chemical inhibitors and antibodies. Recently, S-FLU viruses coated in avian H7 hemagglutinins (15) and the glycoprotein from Ebola viruses (16, 17) have been evaluated as safe and accurate pseudotypes for assaying inhibition of cell entry. This enables the characteristics of coat proteins from highly virulent viruses to be investigated in biosafety containment level 1 or 2 rather than in high-containment facilities.

S-FLU viruses have shown promise as broadly protective vaccines in mice, ferrets, and pigs. They can be administered into the lung intranasally (mice and ferrets) or by aerosol (pigs) without causing pathology. Heterologous protection is induced in the absence of a neutralizing antibody response to hemagglutinin at the low doses administered to the lungs. Protection is associated with strong T-cell responses, including CD8+ T-cell responses in the lungs (7), and a strain-specific antibody response to neuraminidase, which reduces challenge virus titers in the respiratory tract in mice (13, 15) and ferrets (4, 18) and may (19) or may not (18) reduce challenge virus titers in pigs. No sterile immunity is evoked in any model species following administration of S-FLU to the lung. While S-FLU viruses protect against severe disease, they therefore are not able to prevent infection, even if they are perfectly antigenically matched to the challenge strain.

In this study, we explore whether expressing hemagglutinin epitopes from a non-functional hemagglutinin molecule instead of S-HA or enhanced green fluorescent protein (eGFP) in the S-FLU expression cassette drives their cell surface expression and stimulates neutralizing antibody responses without negatively impacting on the existing immunogenicity and single-cycle nature of S-FLU viruses.

A multitude of single or double nucleotide mutations, which significantly ablate or abolish hemagglutinin fusion function, have been described previously. While alone these mutations generate single-cycle viruses with a high rate of reversion to infectivity, using them in combination could allow for the generation of a full-length, rationally designed, multi-mutated hemagglutinin molecule, which is irreversibly non-functional but antigenically preserved.

Here, we describe the iterative design of a non-functional multi-mutated hemagglutinin immunogen named CLEARFLU. We show that single-cycle influenza viruses expressing CLEARFLU hemagglutinin generate neutralizing antibody responses as well as broadly reactive T-cell responses in mice, when administered at low dose to the lungs.

## RESULTS

### CLEARFLU design and expression

The design of CLEARFLU hemagglutinins was refined through three iterations using H1, H3, and H7 hemagglutinins (Table 1). Each CLEARFLU design included seven independent sets of mutations known to block or heavily ablate hemagglutinin fusion activity, acting through up to five different mechanisms as follows: resistance to proteolytic cleavage (20, 21), conformation locking through disulfide bonding (22, 23), receptor inactivation (24, 25), inhibition of the fusion peptide (26), and B-loop inactivation (27).

**TABLE 1:** Mutations introduced to inactivate wild-type hemagglutinin genes in each version of the CLEARFLU design

Mutation type	Mutation position (H3 numbering system [28])	Rationale	CLEARFLU version^*b*^		
			1	2	3
**C**leavage resistance	HA1-**R329Q^*a*^**	Cleavage of the precursor HA0 into HA1 and HA2 by trypsin is necessary for conformational changes required for cell entry. Viruses with mutations at the cleavage site cannot replicate due to changes in protease sensitivity (20, 21)	✓	✓	✓
**L**ocked by inter-monomer disulfide bonds	Head: HA1-**212C** and HA1-**216C**	The introduction of inter-chain disulfide bridges between head regions prevents membrane fusion for cell entry (22). Disulfide bridges between stem regions increase the stability of the hemagglutinin trimer and may prevent fusion (23)	✓	✓	✓
	Stem: HA1-**30C** and HA2-**47C**		✓	✓	✓
**Re**ceptor binding abolished	HA1-**Y98F**	The change from tyrosine to phenylalanine prevents the formation of a hydrogen bond with sialic acid residues necessary for optimum binding (24) and attenuates influenza in mice, but has a high rate of back-mutation (25)	✓	✓	
**F**usion peptide inhibited	HA2-**G1Q** HA2-**L2G**	Mutations in the fusion peptide abolish fusion. The residues that are most highly conserved are most likely to block function when mutated (26)	✓	✓	✓
	HA2-**W14A** HA2-**W21A**		✓		
	HA2-**I6G** HA2-**G8A**			✓	✓
B **L**oop inactivation by proline residues	HA2-**F63P** and HA2-**F70P**	Introducing two proline residues in the B loop prevents conformational changes needed for fusion (27)			✓

^*a*^ Bold in column 1 emphasizes the letters contributing to the "CLEARFLU" name, and bold in column 2 emphasizes the mutation names as referred to in this paper.

^*b*^ "✓" indicates that the mutation is included in this version of the design.

**Fig 1:**
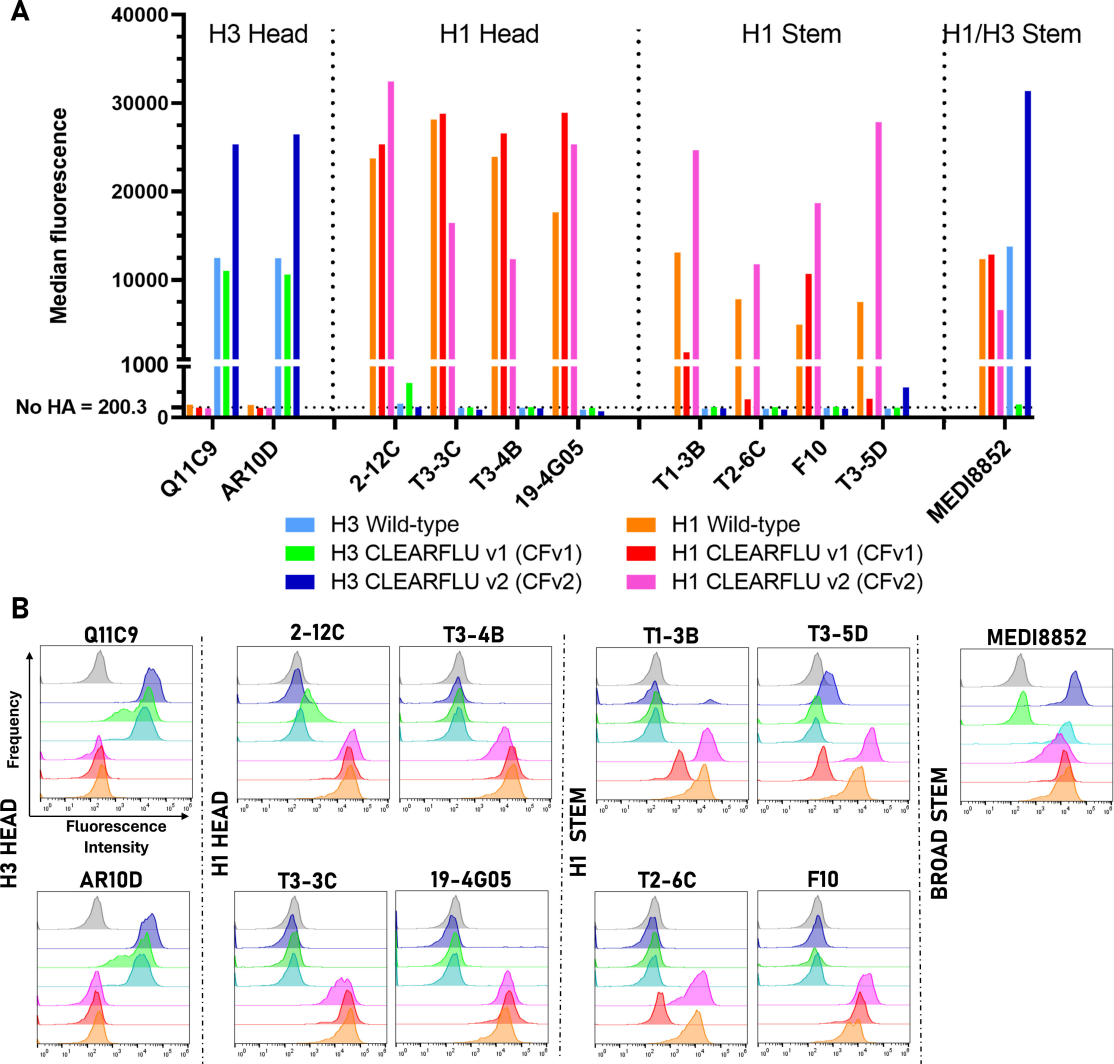
Antibody binding panel to CLEARFLU versions 1 and 2 expressed in stably transduced MDCK–SIAT1 cells. MDCK–SIAT1 cells were transduced to express H1 A/England/195/2009 and H3 A/Hong Kong/5738/2014 CLEARFLU and wild-type hemagglutinins and sorted for high expression with antibodies T3-3C (29) (**H1**) or AM3C (**H3**) (produced in-house, manuscript in preparation). Hemagglutinins were bound with head or stem-targeted human primary antibodies, and binding was detected with a FIT-C-labeled goat anti-human IgG secondary antibody. (A) Bar graph showing median fluorescence. The average median fluorescence for three populations of unstained cells, which were not transduced to express hemagglutinin is shown as the no-HA control. (B) Histogram plots showing the relative frequency of cells detected with a given fluorescence intensity. Gray plots show unstained cells, which were not transduced to express hemagglutinin.

In the first design, the selected mutations disrupted binding by some antibodies (T1-3B, T3-5D, T2-6C [29] and MEDI8852 [30]) to the stem of H1 and H3 hemagglutinins expressed in cell lines (Fig. 1). This binding was restored for CLEARFLU version 2 by reverting mutations W14A and W21A to wild type and replacing these with I6G and G8A, which also inactivate the fusion peptide but are closer to the N terminus and do not interfere with the binding site for these antibodies. Antibody binding to the hemagglutinin globular head did not appear to be affected by the introduction of any candidate mutations.

**Fig 2:**
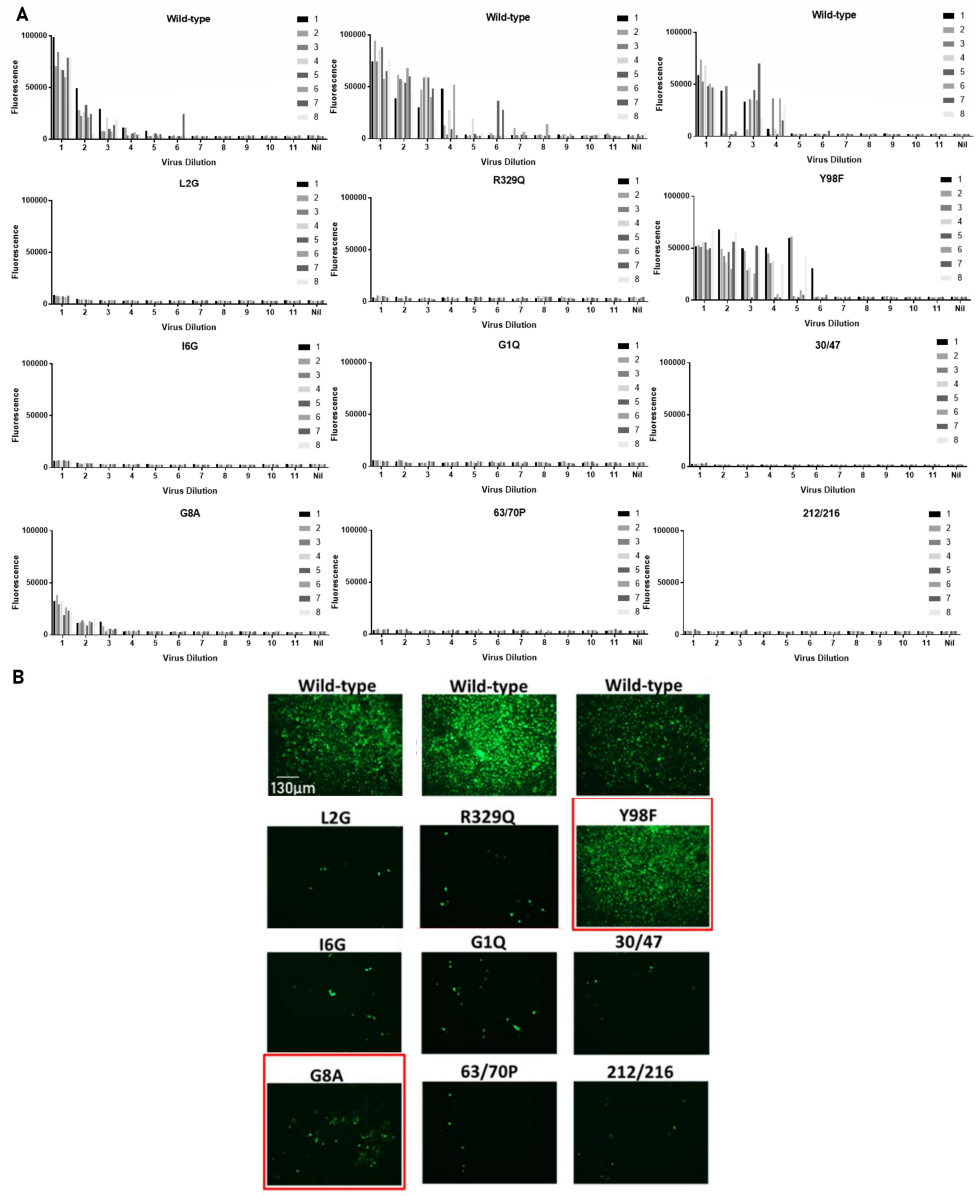
Replication of S-FLU virus in cell lines expressing hemagglutinin with a single CLEARFLU candidate mutation. MDCK–SIAT1 cells were transduced to express the selected mutant H7 A/Hong Kong/125/2017 hemagglutinin and sorted for high expression with H7 antibody 4A14 (33). Cells were infected with an eGFP-expressing S-FLU virus pseudotyped with functional hemagglutinin in half-log dilutions, starting at 1,000 50% tissue culture infectious dose (TCID50) per well. Eight replicates were used at each dilution. The experiment was split into three sets, each of which was repeated with the same results. Virus used for infection: H7N1 S-FLU [S-eGFP/N1(A/Puerto Rico/8/1934) coated in H7(A/Netherlands/219/2003)]. (A) Bars show the fluorescence of each well in a 96-well plate after 48 h. (B) Fluorescence microscopy images show cells in dilution 1 expressing eGFP after S-FLU infection, with clusters of infected cells indicating virus replication.

To verify that the mutations in the CLEARFLU version 2 design did indeed inactivate hemagglutinin fusion activity, we tested the replication of S-FLU viruses in cell lines expressing H7 A/Hong Kong/125/2017 hemagglutinins each with a single inactivating mutation (see Fig. 2). As S-FLU viruses express eGFP instead of hemagglutinin, green comma-shaped plaques of infected cells are formed only in cell lines expressing functional hemagglutinin. We found no evidence that the Y98F mutation previously described to inactivate the sialic acid binding site reduced the growth of S-FLU in H7 transduced MDCK–SIAT1 cells. This supports previous observations by others that Y98F leads to reduced receptor binding, but only limits infection in cells with low sialic acid expression (25, 31). As MDCK–SIAT1 cells overexpress α-2,6-sialic acids, this effect was not seen here (32). The G8A mutation appeared to significantly reduce the size of viral plaques, and we did not observe peaks of fluorescence in middle dilutions to suggest efficient expansion of single viral clones. This suggests that the intermediate fluorescence recorded for G8A is due to many viruses replicating poorly and that G8A reduces, but does not abolish, fusion activity in this context. All the other mutations appeared to completely abolish hemagglutinin-dependent replication.

**Fig 3:**
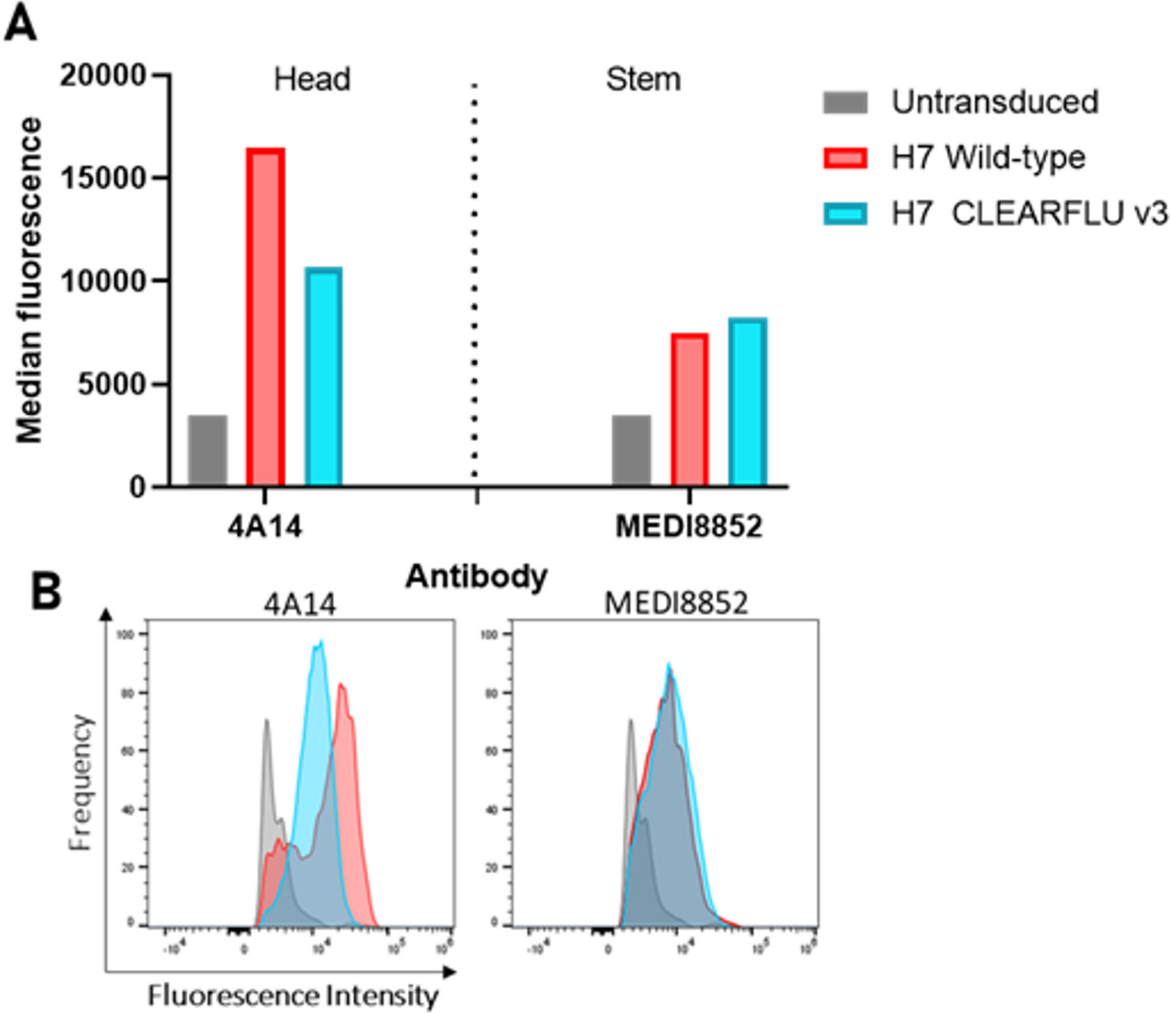
Antibody binding to CLEARFLU version 3 expressed in stably transduced MDCK–SIAT1 cells. MDCK–SIAT1 cells were stably transduced to express H7 A/Hong Kong/125/2017 CLEARFLU version 3 hemagglutinin and sorted for high expression with H7 antibody 4A14. Hemagglutinins were bound with head- or stem-targeted human primary antibodies, and binding was detected with an Alexa Fluor 647-labeled secondary antibody. (A) Bar graph showing median fluorescence. (B) Histogram plots showing the relative frequency of cells detected with a given fluorescence intensity.

To further refine the design of CLEARFLU, we removed the Y98F mutation to avoid disrupting untested neutralizing epitopes around the receptor binding site. To maintain the number of inactivating mutations, we introduced two proline residues at positions 63 and 70 in HA2, which together prevent conformational changes in the B loop required for fusion (27) and are not permissive of S-FLU replication (Fig. 2). CLEARFLU version 3 thus contains an intact receptor binding site (Y98), seven fully inactivating mutation sets (R329Q, G1Q, L2G, I6G, 30/47C, 212/216C, 63/70P), and the partially attenuating mutation HA2 G8A. An antibody panel specific for H7 hemagglutinin (33) confirmed that this combination of mutations did not disrupt protein folding (Fig. 3).

### Genomic stability of CLEARFLU viruses

Viruses expressing H7 A/Hong Kong/125/2017 CLEARFLU version 3 in the hemagglutinin expression cassette were generated by multiplasmid transfection of HEK 293T cells (9). These viruses were expanded in MDCK–SIAT1 cells stably expressing H1 A/Puerto Rico/8/1934 functional hemagglutinin.

**Fig 4:**
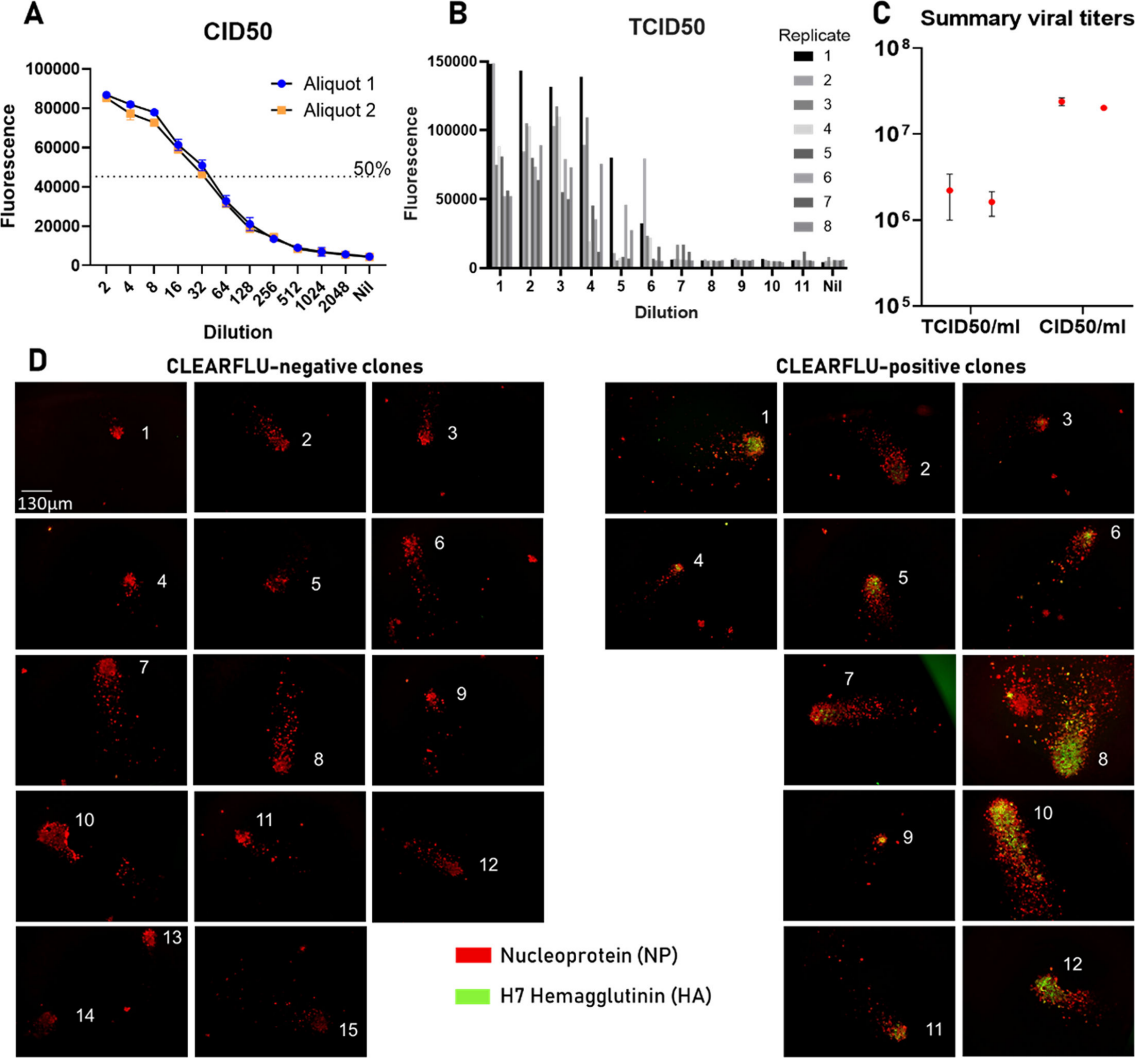
Titration and hemagglutinin expression of CLEARFLU version 3 viruses. (A–C) Two aliquots of CLEARFLU version 3 virus reference stock were titrated by CID50 and TCID50 using antibody 2–8C (13) specific to nucleoprotein and an Alexa Fluor 647-labeled secondary antibody. Error bars show the standard deviation around the mean from duplicates. Plot B is representative of two titrations for each aliquot, the results of which are presented in (C). (D) Fluorescence microscopy images show the expansion of CLEARFLU version 3 virus clones in MDCK–SIAT1 cells transduced to express PR8 hemagglutinin. Cells were infected as per TCID50 protocol but were stained after 24 h with primary antibodies 2–8C (biotin conjugated) and 4A14 and then with secondary layers Alexa Fluor 647 streptavidin and goat anti-human Alexa Fluor 488. Clones were defined as clusters of nucleoprotein-stained cells, often with comet-like trails.

CLEARFLU viruses grew to titers >10E6 50% tissue culture infectious dose (TCID50)/mL />10E7 cell infectious dose (CID50)/mL (see Materials and Methods), but while CLEARFLU was strongly displayed on the surface of some infected cells, many viral plaques did not stain with H7-specific antibody 4A14 (33), which targets the receptor binding site (Fig. 4). This suggested that CLEARFLU HA expression had been lost or that the receptor binding site had been disrupted. The loss of full-length CLEARFLU during expansion may not be surprising given that CLEARFLU expression is not expected to confer any replicative advantage in these conditions.

Previous work in our laboratory has shown that S-FLU viruses grow relatively poorly in MDCK–SIAT1 cells expressing Ebola glycoprotein (GP) (16). We therefore trialed expanding CLEARFLU viruses in cells expressing GP to determine whether this would select for expression of the intact hemagglutinin receptor binding site in CLEARFLU version 3 by providing an efficient sialic acid binding function.

**Fig 5:**
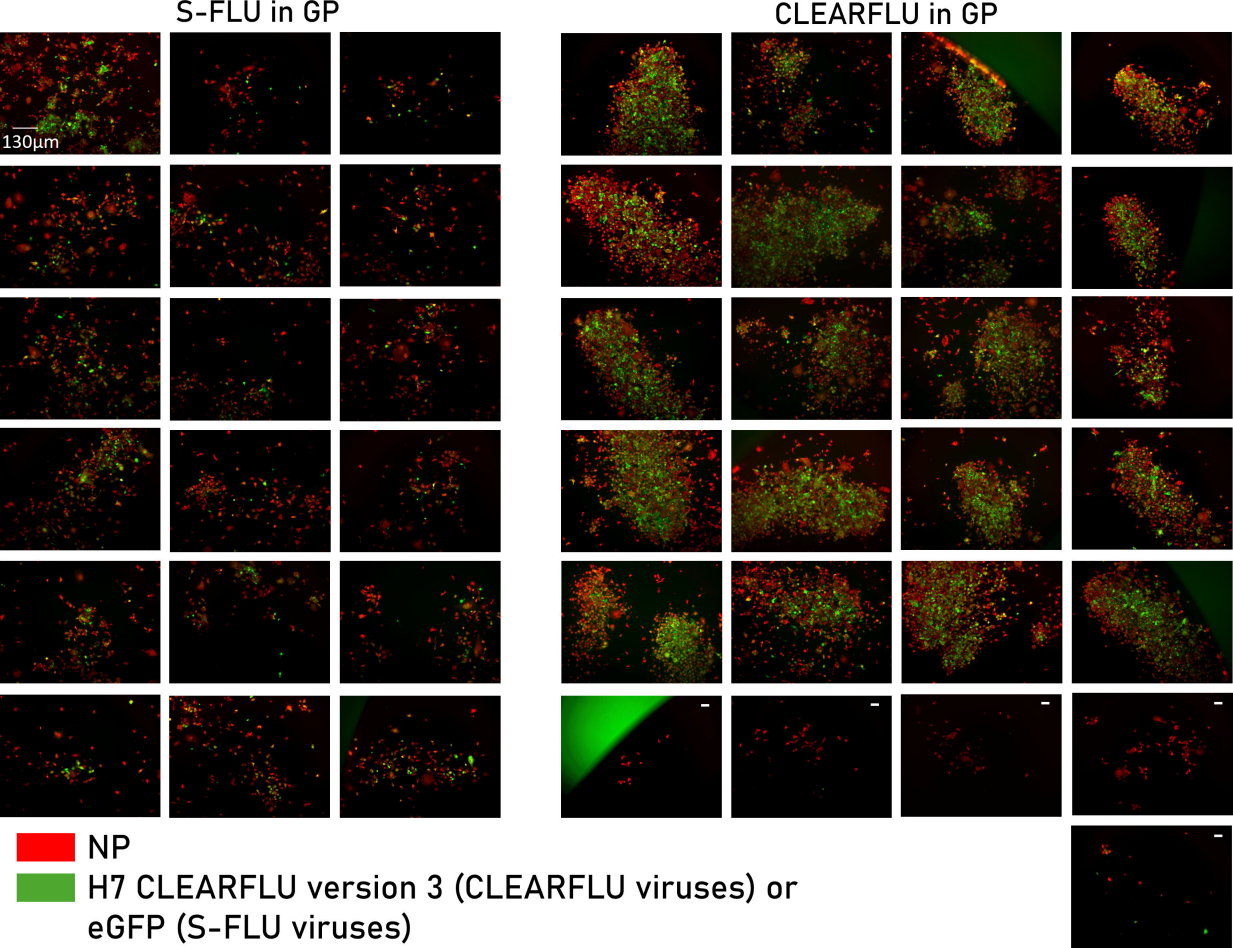
Plaque morphology of CLEARFLU version 3 viruses grown in cells expressing Ebola glycoprotein (GP). Cells were infected with S-FLU or CLEARFLU version 3 virus as per TCID50 protocol and stained after 48 h with anti-nucleoprotein mouse antibody AA5H and an Alexa Fluor 647 secondary antibody (red). Cells infected with CLEARFLU version 3 virus were also stained with H7 hemagglutinin human antibody 4A14 and an Alexa Fluor 488 secondary antibody (green). Fluorescence microscopy images were taken of plaques indicative of clonal expansion. CLEARFLU virus plaques that did not stain with 4A14 are indicated with a white dash. Viruses: H7N1 S-FLU ([S-eGFP/N1(A/Puerto Rico/8/1934)] coated in H7 A/Hong Kong/125/2014) and H7 A/Hong Kong 125/2014 CLEARFLU version 3 reference stock.

**Fig 6:**
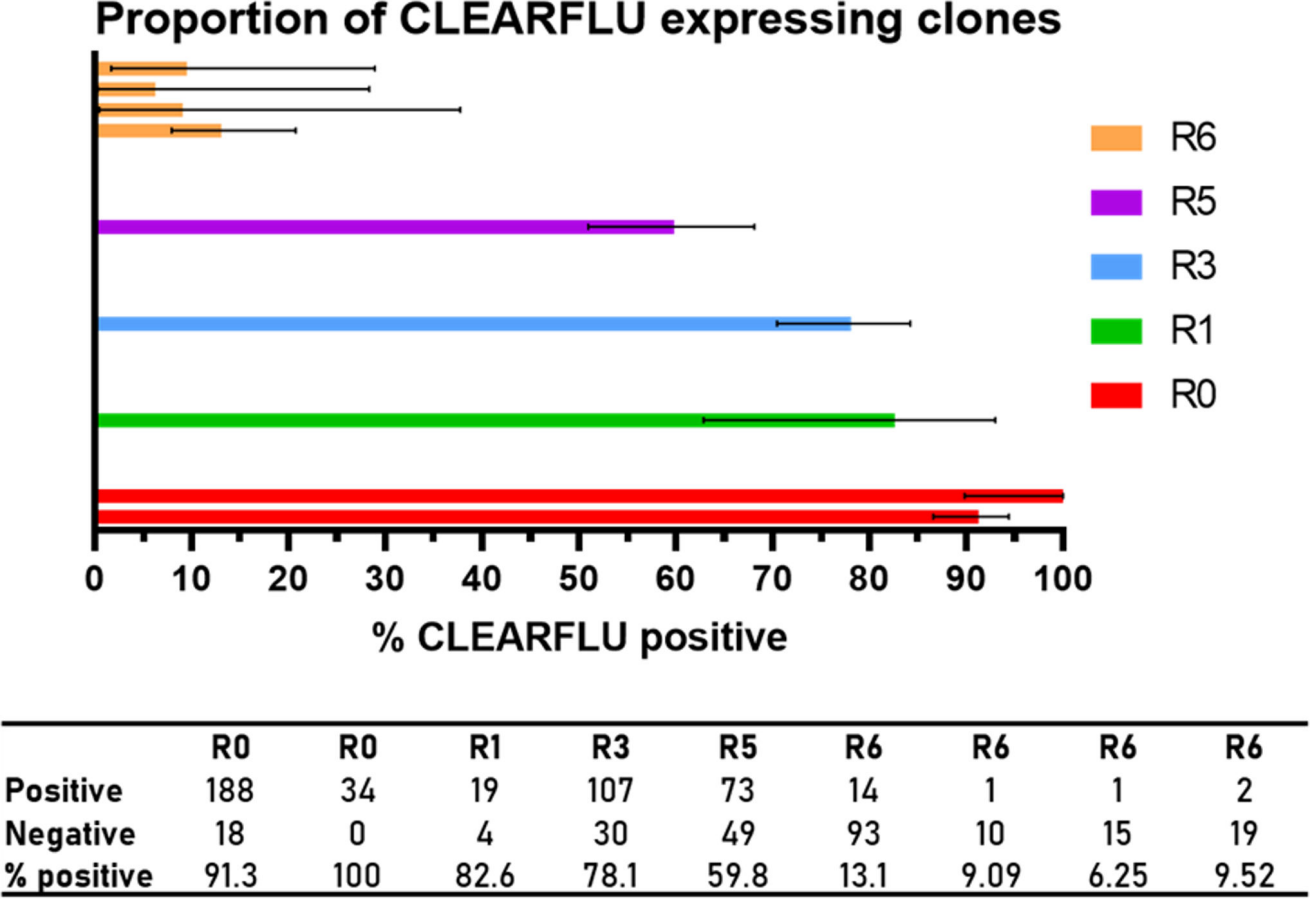
Proportion of clones expressing CLEARFLU version 3 hemagglutinin after passage at low MOI in MDCK–SIAT1 cells expressing Ebola glycoprotein. CLEARFLU version 3 reference stock was used to infect a large flask of MDCK–SIAT1 cells expressing Ebola GP at a multiplicity of infection of approximately 0.01 by TCID50 and then passaged at a 1 in 1,000 dilution every 48 h six times. Aliquots from each round were used to infect MDCK–SIAT1 cells expressing GP in limiting dilution, and viral clones were identified by nucleoprotein staining with AA5H (Alexa Fluor 647 secondary). Clones were scored as positive for CLEARFLU expression if they also stained with 4A14 (Alexa Fluor 488 secondary). Each bar shows an independent count. Error bars show 95% confidence intervals calculated using the Wilson/Brown method for proportions.

CLEARFLU viruses indeed grew to higher titers and formed denser plaques in MDCK–GP cells than S-FLU viruses, and plaques produced by CLEARFLU viruses, which did not stain with hemagglutinin antibody, were similar in morphology to the small diffuse plaques produced by S-FLU viruses (Fig. 5). We tested this apparent selective pressure and genomic stability under these conditions by passaging the virus at low multiplicity of infection (MOI) six times in MDCK–GP cells, representing a 4 × 10^18^ expansion by round six. After six rounds, CLEARFLU viruses still grew to a high titer, and we were able to detect CLEARFLU expression; however, the proportion of clones that stained with 4A14 decreased from over 90% to approximately 10% (Fig. 6). Remarkably, sequencing revealed that no mutations were identified in the coding sequences of any core viral genes in the round six culture.

In isolating viral clones from the round six culture, we were able to identify several CLEARFLU viruses that replicated efficiently to form dense plaques despite *not* staining with the Mab 4A14. Four of these clones were sequenced, and all were found to carry the T148A mutation (N2 numbering; T132A in N1 numbering) in their neuraminidase. The T148A substitution has been previously reported to disrupt a conserved site for N-linked glycosylation in the B1L23 loop [HS**NGT**VKDR] close to the active site of the enzyme (34).

**Fig 7:**
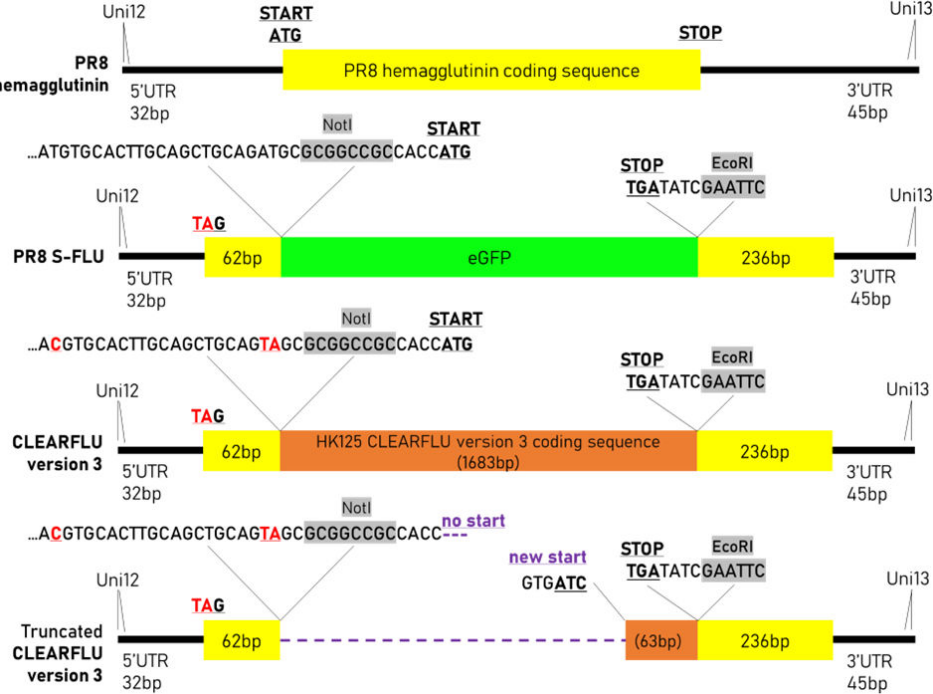
Design of the CLEARFLU version 3 viral expression cassette and truncation after repeated passage in MDCK–SIAT1 cells expressing Ebola glycoprotein. The CLEARFLU expression cassette contains intact packaging signals from the S-FLU expression cassette, which in this design differs from that described in Reference (13) by the alteration of two additional interfering ATG codons upstream of the NotI site. After six rounds of passage at low multiplicity of infection in MDCK–SIAT1 cells expressing Ebola glycoprotein, two viral clones expressed a truncated CLEARFLU construct containing only 63 base pairs of coding sequence flanked by intact PR8 untranslated regions (UTRs).

To confirm that this substitution was responsible for the enhanced growth in these conditions, we introduced the T148A mutation alone into S-FLU. We showed that this mutation enables the neuraminidase to agglutinate red blood cells and enhances cell entry in the GP-transduced MDCK–SIAT1 cells in an oseltamivir and neuraminidase antibody-sensitive manner (see Fig. S12 and S13 in the supplemental material). This suggested that T148A enhanced cell entry by enhancing the binding to sialic acid, possibly reducing the selection for expression of CLEARFLU. Sequencing of the CLEARFLU HA segment of two T148A carrying clones revealed a large truncation of 1,620 nucleotides, leaving a 438 nucleotide fragment containing intact packaging sequences but only 63 nucleotides of CLEARFLU coding sequence (Fig. 7). A full sequence and alignment of the truncated construct are available in Fig. S10 and S11 in the supplemental material. In another clone, which grew well, stained with 4A14, and did not contain the T148A mutation, the CLEARFLU segment remained full length and contained no mutations.

These results showed that expressing the CLEARFLU HA can provide a growth advantage (presumably by enhancing sialic acid binding) to viruses propagated in cell lines transduced with Ebola GP to provide a fusion function. However, substitution of NA T148A can also provide enhanced sialic acid binding, and viruses with this mutation compete with those expressing CLEARFLU HA.

### Murine immune response to CLEARFLU viruses

As the immune response to S-FLU viruses is known to protect mice, ferrets, and pigs against heterologous challenge, we compared the immune response to CLEARFLU and S-FLU viruses in mice. In our first experiment, we assessed the serum antibody response to viruses expressing seasonal H1(A/England/195/2009) and H3(A/Hong Kong/5738/2014) CLEARFLU version 2 hemagglutinins. In the second, we compared local and systemic, humoral and cellular immune responses to a virus expressing H7(A/Hong Kong/125/2017) CLEARFLU version 3 hemagglutinin to those generated by a strain-matched S-FLU.

**Fig 8:**
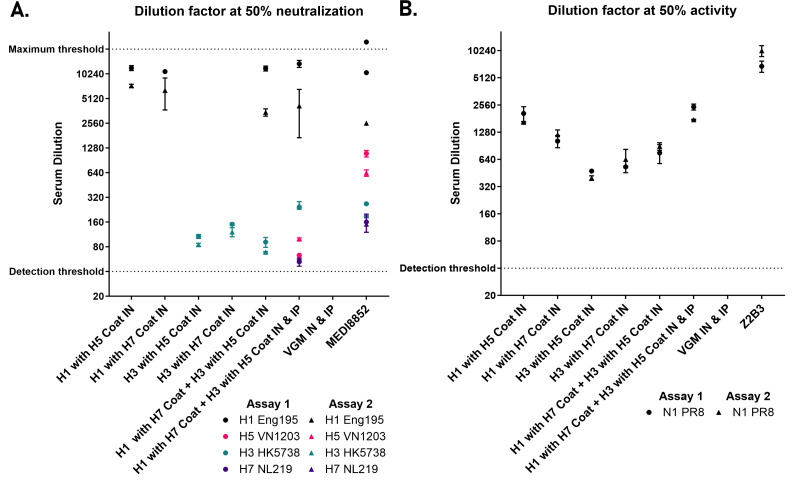
Murine serum antibody response following immunization with CLEARFLU version 2. Serum antibody response in mice immunized with H1 or H3 CLEARFLU version two viruses pseudotyped with H5 or H7 coats. Mice were immunized with 2E6 CID50 virus intranasally (IN), or 1E6 CID50 intranasally and 1E7 CID50 intraperitoneally (IP) twice 28 days apart. Sera were harvested 14 days after the second dose for characterization by microneutralization (**A**) and enzyme-linked lectin assay (**B**). Sera from five mice were pooled for each group, except the viral growth medium (VGM) only control group, for which four mice were used. Error bars show the standard deviation from within-assay duplicates, with results from two independent assays shown. (A) Neutralizing antibody response to H1, H3, H5, and H7 S-FLU by immune mouse sera or control antibody MEDI8852. (B) Inhibition of N1 neuraminidase activity by mouse immune sera or control antibody Z2B3.

**Fig 9:**
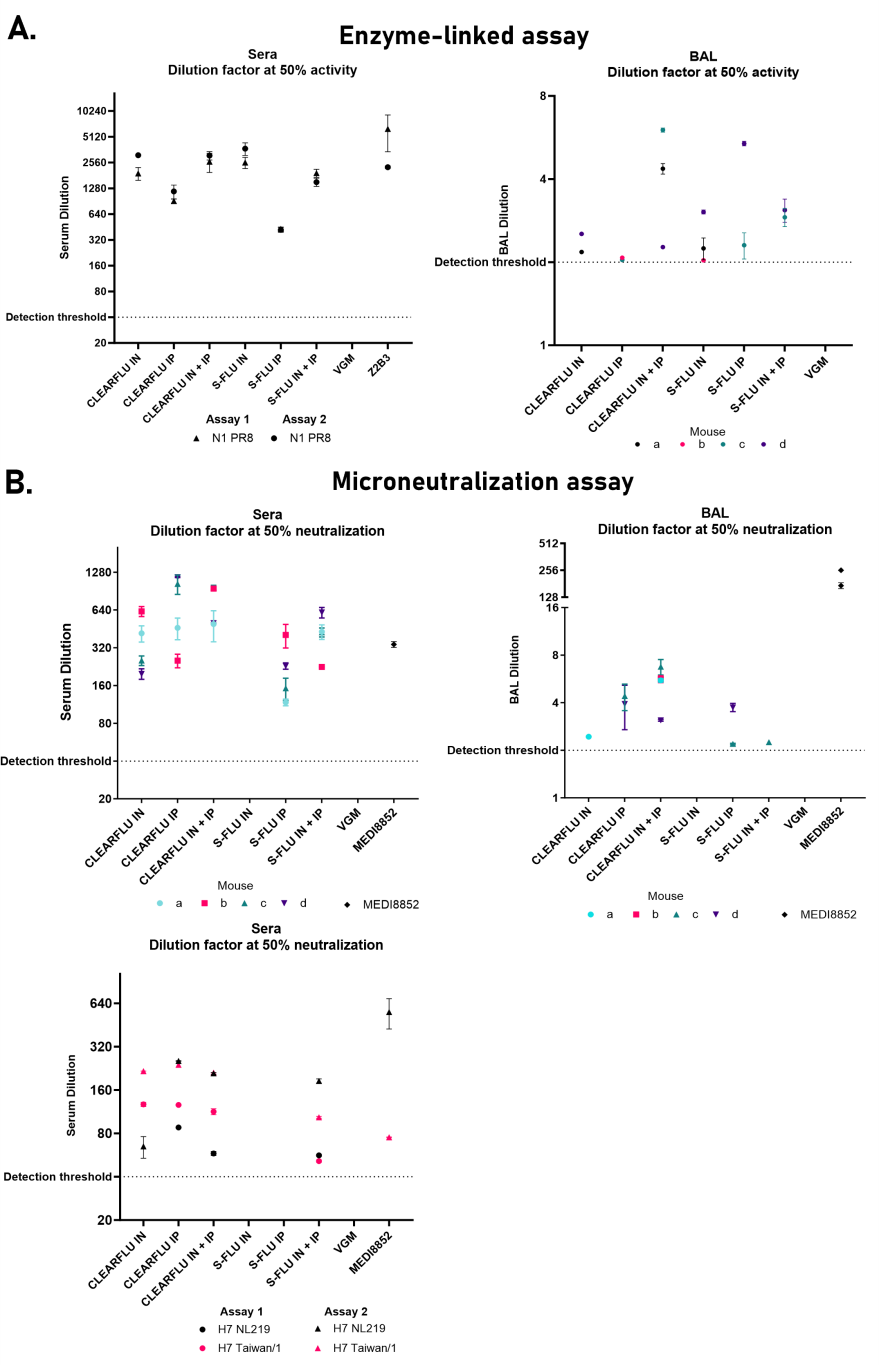
Murine serum and BAL antibody response following immunization with CLEARFLU version 3. Serum and BAL antibody response in mice immunized with H7 CLEARFLU version 3 pseudotyped with an H1 (PR8) coat or a strain-matched H7 S-FLU. Mice a–d in each group were immunized twice 14 days apart with 1E5 TCID50 virus intranasally (IN), 1E6 TCID50 intraperitoneally (IP) or 1E5 TCID50 IN and 1E6 TCID50 IP. Sera and BAL were harvested 68 days after the second dose for characterization by enzyme-linked lectin assay (**A**) and microneutralization (**B**). Error bars show the standard deviation from within-assay duplicates when both duplicates fell above the detection threshold. (A) Inhibition of N1 neuraminidase activity by mouse immune sera, BAL, or control antibody Z2B3. Sera from the four mice were pooled for each group. BAL could not be collected from mouse b in the CLEARFLU IN + IP group, mouse a in the S-FLU IN + IP group or mouse a in the VGM group. (B) Neutralizing antibody response to H7 S-FLU by immune mouse sera, BAL, or control antibody MEDI8852. The top two plots show neutralization of strain-matched A/Hong Kong/225/2017 S-FLU by individual mouse sera or BAL. BAL could not be collected from mouse a in the S-FLU IN + IP group or mouse a in the VGM control group. The bottom plot shows neutralization of unmatched H7 A/Netherlands/219/2003 and A/Taiwan/1/2017 S-FLU by pooled immune sera.

Like S-FLU, CLEARFLU versions 2 and 3 viruses can be safely administered to mice both intranasally and intraperitoneally. We found that CLEARFLU viruses offer an advantage over S-FLU viruses in that they produce a strong neutralizing antibody response to hemagglutinin even when administered to the airways (Fig. 8 and 9). This response was particularly strong for H1 and H7 hemagglutinins compared to H3. For CLEARFLU version 3, this neutralizing response was strongest in the serum, but also detected in bronchoalveolar lavage (BAL) and, while subtype-specific, showed some within-subtype cross-neutralization in the serum (Fig. 9). The antibody response to the neuraminidase in the BAL and serum was similar to that generated by S-FLU (15).

**Fig 10:**
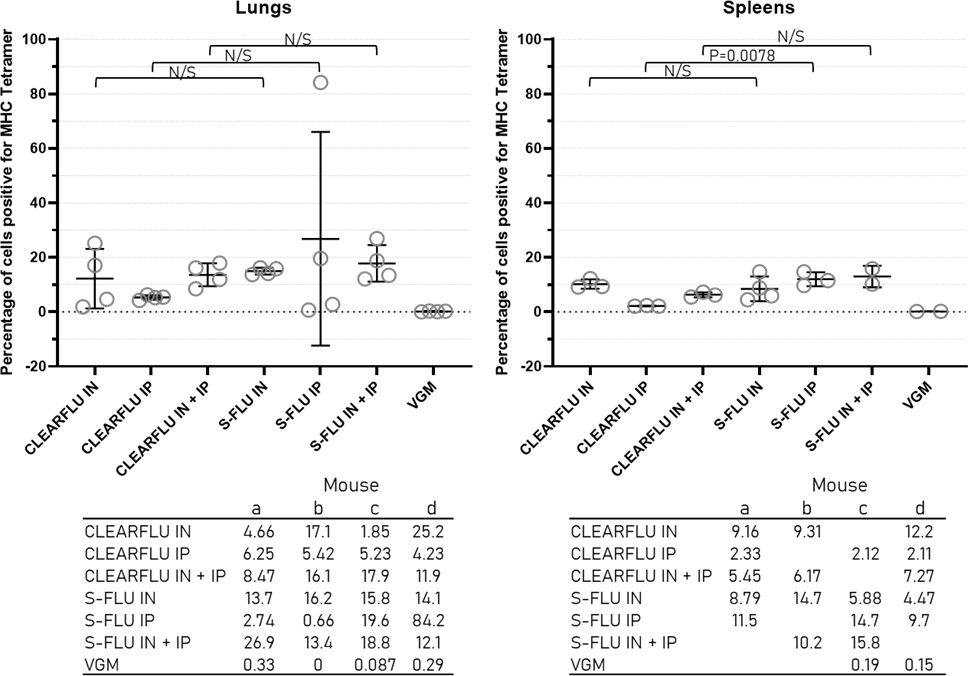
Murine T-cell response to CLEARFLU version 3. Mice were immunized as described in Fig. 9. Circles indicate the percentage of CD8+ T cells positive for MHC tetramer in the lungs and spleen of each mouse a—d in each group. Bars show the mean and standard deviation for each group. For spleens, data could not be collected for one individual in the CLEARFLU IN, CEARFLU IP, CLEARFLU IN + IP, and S-FLU IP groups and for two individuals in the S-FLU IN + IP and VGM control groups. Multiple t tests were used to compare CLEARFLU and S-FLU groups for each administration method. The Holm–Sidak method was used to correct for multiple comparisons. All differences were non-significant at the *P* = 0.05 level except for IP groups in the spleen where *P* = 0.0078.

The CLEARFLU version 3 virus generated CD8+ T-cell responses to nucleoprotein in the lungs and spleen comparable to a strain-matched S-FLU, which have previously been shown to protect against heterologous—including heterosubtypic—challenge in mice and ferrets and partially protect pigs (Fig. 10).

Overall, these results indicate that CLEARFLU and S-FLU viruses have very similar immunogenicities in mice, with the notable exception that the expression of CLEARFLU hemagglutinins on the surface of cells is able to generate a strong neutralizing antibody response after intranasal delivery at low dose, and may therefore provide sterile immunity against strain-matched challenge.

## DISCUSSION

We have described a methodology for designing hemagglutinins inactivated with multiple mutations, which can be safely expressed as part of single-cycle viruses. We have shown that this approach can be applied to both group 1 (H1) and group 2 (H3 and H7) hemagglutinins without altering expression or antigenicity. In doing so, we have developed a promising vaccine platform for stimulating a combination of broad local T-cell responses and strain-specific neutralizing antibodies after intranasal administration. The alternative way to induce neutralizing antibody with S-FLU is to combine intranasal administration with a larger dose in the periphery (13, 35). A single administration is clearly preferable.

CLEARFLU hemagglutinins expressed within the S-FLU expression cassette were displayed on the cell surface and immunogenic in mice. Although we identified that some CLEARFLU expression is lost over time during expansion of viral stocks, both viral titer and the proportion of viral clones still expressing CLEARFLU remained high for five rounds of expansion, allowing large quantities of virus to be produced without the need for recloning. While after expansion some viral clones expressed a truncated CLEARFLU segment, we found no mutations in viral clones with a full-length segment, indicating that the CLEARFLU segment was relatively genetically stable. Truncation of the CLEARFLU segment appeared to be associated with a T148A mutation in the neuraminidase, which likely enabled neuraminidase-dependent sialic acid binding and enhancement of cell entry. CLEARFLU viruses overall appeared to be genetically stable, as no mutations were identified elsewhere in the viral genome.

Hemagglutinin-inactivated single-cycle influenza viruses, like CLEARFLU, could be suitable as pre-pandemic as well as seasonal vaccines as they do not encode a viable hemagglutinin that could theoretically reassort with seasonal viruses. The presence of multiple independent mutations scattered throughout the hemagglutinin renders negligible the probability of reversion to pathogenicity and reduces the already low risk of homologous recombination with wild-type hemagglutinins to yield functional new variants (36). As hemagglutinin is a key contributor to antigenic shift, it is particularly beneficial to prevent this segment from acting as a donor sequence for wild-type viruses. Thus, an S-FLU expressing a CLEARFLU version 3 H7 hemagglutinin would be theoretically safer than a LAIV encoding a viable H7 sequence (37, 38) that could, in principle, reassort with a seasonal influenza.

Moreover, as the pseudotyping hemagglutinin is the determinant of cell entry but not the antigen driving neutralizing antibody responses after intranasal dosing, pseudotyping cell lines can be selected to avoid pre-existing immunity while maximizing sialic acid binding. Meanwhile, the multi-mutated CLEARFLU hemagglutinin can be chosen entirely for its immunogenicity. This could be a key advantage over other approaches, like LAIV, where pre-existing immunity reduces vaccination efficiency against seasonal influenza.

Unlike for many single-cycle viruses, which lack all or a large part of a viral gene, the full complement of viral antigens should be expressed in RNA, protein,and peptide form in the appropriate cellular location after immunization with CLEARFLU, maximizing immunogenicity. In particular, neutralizing antibody responses to both hemagglutinin and neuraminidase are desirable as they act independently (39) to limit or abolish virus shedding and onward transmission (40), reduce the risk of pathology in individuals with weak T-cell responses, and restrict the opportunity for wild-type virus evolution. CLEARFLU, therefore, represents a clear advantage over S-FLU and other intranasal vaccine candidates, which do not induce neutralizing antibody.

## MATERIALS AND METHODS

### Experimental models

MDCK–SIAT1 cells generated by transducing MDCK cells with human 2,6-sialyltransferase 1 (SIAT1) to express higher levels of sialyl-α2,6-galactose moieties (32) were obtained from the European Collection of Authenticated Cell Cultures (ECACC 05071502). Human Embryonic Kidney (HEK) 293T cells (HEK cells stably transduced to express SV40 Large T antigen) were obtained from the Sir William Dunn School of Pathology, Oxford University (Ervin Fodor). All cells were grown in D10 (10% FCS + 2 mM L-glutamine + 100 U/mL penicillin + 100 µg/mL streptomycin in DMEM) at 37°C with 5% CO_2_ and passaged when confluent. MDCK–SIAT1 cells were harvested by incubating with Trypsin-EDTA for 10 min and centrifuging in 50 mL of D10. 293T cells were harvested using 2 mM EDTA in phosphate-buffered saline (PBS) and spun in 50 mL PBS. Pellets were resuspended in D10 for passaging, viral growth medium (VGM) for viral assays, or at approximately 10 million cells/mL in freezing medium for storage.

C57BL/6 and BALB/c female mice were obtained from Envigo RMS Inc, Bicester and maintained in individually ventilated cages at the Biomedical Services facility at the John Radcliffe Hospital, Oxford. They were 6–8 weeks old at the start of experiments.

### Molecular studies

CLEARFLU hemagglutinins were designed by incorporating inactivating mutations into wild-type hemagglutinin sequences from A/England/195/2009 (H1), A/Hong Kong/5738/2014 (H3), and A/Hong Kong/125/2017 (H7). DNAs were human codon optimized. Restriction enzyme cloning sites for NotI and EcoRI were added to either end of the sequence to facilitate cloning into vector pHR-SIN or the S-FLU cassette for mammalian or viral expression, respectively. All sequences were ordered from GeneArt and resuspended in 50 µL 10 mM Tris/0.1 mM EDTA. Sequences are available in the supplemental material (Fig. S1–S11).

For subcloning into pHR-SIN (39) or the S-FLU expression cassette, 2 µg of GeneArt plasmid was digested using EcoRI and NotI in NEBuffer 3.1. Digestion products were run on gels containing 0.7% agarose and 0.5 µg/mL ethidium bromide in TAE buffer. Samples were loaded using Orange G gel-loading dye. Bands were imaged and extracted under UV light and purified using a QIAquick Gel Extraction Kit from Qiagen according to the manufacturer’s protocol. Inserts were ligated into new vectors in a 30-µL reaction containing 1.5 µLT4 DNA ligase, 3 µL T4 DNA ligase buffer, 3 µL pre-digested and phosphatase-treated vector, and 15 µL of insert. The reactions were incubated at room temperature for at least 1 h.

All plasmids were grown in DH5α *E. coli*. 2 µL of plasmid was added to 5 µL of DH5α ultracompetent cells in Eppendorf tubes on ice. After a 40-min incubation, a heat shock was performed for 30 s at 42°C, followed by incubation on ice for 1 min. 1 mL of LB was added before incubating at 37°C for 1 h and then spreading the broth on LB-agar plates containing ampicillin. After overnight incubation at 37°C, colonies were picked into 13 mL LB containing ampicillin for a further overnight incubation at 37°C on a 200-rpm shaking platform. 0.9 mL of the broth was stored at −80°C in Hogness solution for future use. The remaining cells were then pelleted by centrifugation at 3,000 rpm for 45 min and the supernatant removed. Plasmids were extracted from the pellets using a QIAprep Spin Miniprep Kit. To check pHR-SIN plasmids for correct insert length, 20 µL digests with 2 µL plasmid DNA and 0.5 µL each of NotI and EcoRI were set up. Phusion PCR (Thermo Scientific) was performed as recommended by the manufacturer. PCR products were run on 0.7% agarose gels containing ethidium bromide as described for digestion products.

Agilent QuickChange Lightning Site-Directed Mutagenesis kits were used to introduce specific mutations by PCR into the hemagglutinin sequence of A/Hong Kong/125/2017 to generate versions with a single inactivating mutation set. The sequence was subcloned into vector pcDNA3.1− as described above for mutagenesis, and then subcloned into vector pHR-SIN afterward using the same restriction sites. The bacteria provided were grown up in the same conditions as described for DH5α *E. coli*.

### Transfection and transduction of cell lines

Multi-plasmid transfection of HEK 293T cells for generating recombinant viruses was performed using Lipofectamine 2000 (Thermo Fisher) as described previously (9, 13).

MDCK–SIAT1 cells were transduced to stably express hemagglutinin by lentivirus transduction. First, HEK 293T cells were transfected with 1.33 µg each of plasmids containing VSV-G, Gag/Pol and the transgene of interest in a pHR-SIN vector to produce lentivirus (41). After 48 h, the supernatant was incubated with MDCK–SIAT1 cells in the presence of 8 µg/mL polybrene in D10. After 24 h, the lentivirus infection was repeated, and cells were harvested once confluent.

### Staining cells for fluorescence-activated cell sorting (FACS)

Confluent T175 flasks of MDCK–SIAT1 cells were harvested and spun in D10 at 1,400 rpm. The cell pellet was resuspended in 1 mL of 20 µg/mL primary antibody in D10 with 10 mM HEPES and incubated on ice for 1 h. Cells were then washed with 50 mL of D10 and resuspended in 1 mL of 20 µg/mL secondary antibody and incubated on ice for 1 h. Cells were again washed with D10, spun, and resuspended in 7 mL of D10 before being passed through a 0.22-µm syringe filter and sorted using a BD FACSAria III. Sorted cells were grown up and passaged in T175 flasks to produce cell stocks for subsequent experiments.

### Staining cells for analysis by flow cytometry

MDCK–SIAT1 cells were aliquoted into FACS tubes at approximately 1 million cells per tube. The tubes were spun at 1,400 rpm, and the cells were resuspended in 50 µL of 20 µg/mL primary antibody diluted in cold FACS wash (1% FCS + 0.01% azide in PBS). After 1 h at 4°C, 2 mL of FACS wash was added, and the cells were spun and resuspended in 50 µL of 20 µg/mL secondary antibody. After a further hour at 4°C, the cells were washed in FACS wash and resuspended in 300 µL of FACS fixative (1% FCS + 1% formalin in PBS). Cells were analyzed using a CyAn ADP flow cytometer by Beckman or an Attune NxT flow cytometer by Invitrogen. Data were analyzed using FlowJo v10.6.1. Forward and side scatter were used to gate for single cells only.

### Staining cells for plated viral assays

Confluent MDCK–SIAT1 cells in 96-well plates were stained with 40 µL of 1–5 μg/mL primary antibody per well for 1 h at 4°C, washed thoroughly with PBS, and then stained with 40 µL of 5 µg/mL secondary antibody in the same conditions. For staining nucleoprotein, cells were first fixed by incubation with 100 µL of 10% formalin for 30 min at 4°C and then incubated with 100 µL of permeabilization buffer (0.5% Triton X-100) at room temperature for 20 min. For stains not involving nucleoprotein, cells were fixed after staining and were not permeabilized. Double stains were performed by incubating with both primary antibodies—one mouse and one human—simultaneously and then both secondary antibodies, as they do not cross-react significantly. Antibodies were diluted in FACS wash. Fixed cells were stored in PBS. Images were taken with a Zeiss fluorescence microscope using ×10 magnification. Images were managed using Fiji/ImageJ. Fluorescence was quantified using a CLARIOstar microplate reader (BMG Labtech).

### Virus titrations

Isolation of viral clones by limiting dilution and quantification by TCID50 was performed as described previously (13). Briefly, virus was added to 3E4 MDCK–SIAT1 cells in serial half-log dilutions in a 96-well plate in 200-µL volume of VGM with TPCK-trypsin, with eight replicates for each dilution. After 48 h, cells were fixed, permeabilized, and stained as described above, and the dilution at which 50% of wells were infected was calculated using the Reed Muench method (42).

The CID50 was determined by adding 3E4 MDCK–SIAT1 cells to serial twofold dilutions of virus in duplicate rows in a 96-well plate without the addition of trypsin. After overnight incubation, the cells were fixed, permeabilized, and stained, and the dilution of virus at which 50% of cells in a well were infected was determined by linear interpolation.

### *In vivo* work

#### Immunization and sample collection

For intranasal immunization, mice were anesthetized using 4.5% isofluorane, and drops of virus were pipetted onto the nose to be inhaled one by one to a total volume of 50 µL. For intraperitoneal immunization, 500 µL of virus was injected into the intraperitoneal space using a 0.5-mL insulin syringe and a 29G needle. Mice were humanely killed by exposure to slowly rising concentration of CO_2_, and death was confirmed by cervical dislocation. BAL was obtained by nicking the trachea and using a syringe to wash 1 mL of sterile PBS through the lungs three times. Blood was harvested by cardiac puncture using a 23–25G needle and a 1- or 2-mL syringe and stored in a BD microtainer with SST gel for 30 min to allow clotting before spinning at 10,000 rpm for 5 min. Serum was collected from above the interface gel and heat inactivated at 56°C for 30 min. Spleens were cut into small pieces and passed through a 70-µm cell strainer mesh to make single-cell suspensions in R10 (10% FCS + 2 mM L-glutamine + 100 U/mL of penicillin + 100 µg/mL of streptomycin in RPMI-1640). Lungs were minced and treated with 4 mL of enzyme solution (2 mg/mL of collagenase IV + 200 U in RPMI-1640) for 30 min at 37°C before being passed through a 70-µm cell strainer mesh. The suspension was spun at 1,200 rpm for 5 min, and the cell pellets were treated with RBC lysis buffer (Qiagen) for 10 min and then washed with R10. The cell pellets were resuspended in 5 mL of 40% Percoll in a 15-mL tube. A 5-mL layer of 80% Percoll was laid down at the bottom of the tubes using a Pasteur pipet before spinning the cells at 2,000 rpm for 25 min using a slow deceleration rate. Leukocytes were collected from the Percoll interface and washed in R10 twice before being resuspended in R10.

#### Microneutralization assays

Microneutralization assays were performed as described previously (13) with slight modifications. Sera were diluted 1 in 20 before use; BAL samples were not prediluted. For some experiments, sera from animals in the same group were pooled. S-FLU viruses with known neutralization profiles were used as sources of hemagglutinin and titrated before use to ensure maximum signal. Briefly, sera or BAL were set up in serial twofold dilutions in 50 µL of PBS in a 96-well plate and incubated with 50 µL of S-FLU virus for 2 h at 37°C before adding 3E4 MDCK–SIAT cells in 100 µL of VGM. After overnight incubation at 37°C, eGFP fluorescence was read.

#### **Enzyme-linked lectin assays (ELLA**)

Enzyme-linked lectin assays (ELLA) were performed to assay inhibition of neuraminidase activity by immune sera or BAL (43, 44; modified by 15). S-FLU viruses were used as sources of active neuraminidase and selected to avoid interference by neutralizing antibodies to hemagglutinin. Ninety-six-well ELISA plates were coated in fetuin by incubating in 50 µL of fetuin solution overnight at 4°C. Doubling dilutions of sera or BAL were set up in 60 µL, to which 60 µL of S-FLU virus was added for incubation at 37°C for 2 h. Fetuin plates were washed four times with PBS before adding 100 µL of S-FLU and serum/BAL mix to each well and incubating for 18 h at 37°C. After washing four times with PBS, 50 µL of PNA–HRP (peanut agglutinin conjugated to horseradish peroxidase) solution was added for a 1.5-h incubation. After washing four times with PBS, 50 µL of peroxidase substrate solution (OPD) was added, and the plates were developed for approximately 15 min before stopping the reaction with 50 µL of 1 M H_2_SO_4_. Absorbance at 492 nm was read immediately using a CLARIOstar microplate reader.

#### Detection of NP-specific T cells

Approximately 1 million homogenized splenocytes or lung cells were added to each well of a 96-well plate and washed with R10. MHC class I tetramers (NP_366-374_ ASNENMETM labeled with APC) were diluted 1 in 200 in R10, and 200 µL was added to each well. After 30 min at 37°C, the cells were washed with PBS. Of Zombie Violet, 25 µL was used at 1:1,000 in PBS to stain dead cells by incubating in the dark at room temperature for 10 min. Of an antibody mix, 25 µL was added to each well to simultaneously stain for surface markers CD8 (APC-Cy7 53–6.7), CD44 (Alexa Fluor 488 IM7), Cd11b (Brilliant Violet 421 M1/70), and B220 (Brilliant Violet 421 RA3-6B2), with each antibody diluted 1 in 200 in FACS buffer. After 30 min on ice in the dark, the cells were washed with PBS and fixed using 100 µL of cold 5% formalin for 10 min at 4°C, and then washed again and resuspended in PBS. CD8+ T cells positive for MHC tetramer were analyzed using an Attune NxT flow cytometer (Thermo Fisher Scientific) and FlowJo v10.6.1. Ultracompensation beads were used to account for overlapping emission spectra.

## Data Availability

Data generated in this study are available from the corresponding authors on request. The products generated can be made available to other laboratories for research use.

## SUPPLEMENTAL MATERIAL

Supplemental figures jvi.00331-24-s0001.pdf Figures S1 to S13.

ASM does not own the copyrights to Supplemental Material that may be linked to, or accessed through, an article. The authors have granted ASM a non-exclusive, world-wide license to publish the Supplemental Material files. Please contact the corresponding author directly for reuse.
