# Supplementary material for: A locally administered single-cycle influenza vaccine expressing a non-fusogenic stabilized hemagglutinin stimulates strong T-cell and neutralizing antibody immunity
Source: J Virol. 2025 Jan 27;99(2):e00331-24. doi: 10.1128/jvi.00331-24 (PMC11853075; doi:10.1128/jvi.00331-24)

Supplementary material

Fig S.1

A/England/195/2009 wildtype hemagglutinin (human codon-optimised)

ATAAGAATGCGGCCGCCACCATGAAGGCCATCCTGGTGGTGCTGCTGTACACCTTCGCCACCGCC
AACGCCGACACCCTGTGCATCGGCTACCACGCCAACAACAGCACCGACACCGTGGATACCGTGC
TGGAAAAGAACGTGACCGTGACCCACAGCGTGAACATCCTGGAAGATAAGCACAACGGCAAGCT
GTGCAAGCTGAGAGGCGTGGCCCCCCTGCACCTGGGCAAGTGCAATATCGCCGGCTGGATTCTGG
GCAACCCCGAGTGCGAGAGCCTGAGCACCGCCAGCAGCTGGTCCTACATCGTGGAGACAAGCAG
CAGCGACAACGGCACCTGTTACCCCGGCGACTTCATCGACTACGAGGAACTGCGGGAGCAGCTG
AGCAGCGTGTCCAGCTTCGAGCGGTTCGAGATCTTCCCCAAGACCTCCTCCTGGCCCAACCACGA
CAGCAACAAGGGCGTGACAGCCGCCTGTCCTCACGCTGGCGCCAAGAGCTTCTACAAGAACCTG
ATCTGGCTGGTGAAGAAGGGCAACAGCTACCCCAAGCTGAGCAAGAGCTACATCAACGACAAGG
GCAAGGAGGTGCTCGTCCTCTGGGGCATCCACCACCCTAGCACCAGCGCCGACCAGCAGAGCCT
GTACCAGAACGCCGACGCCTACGTGTTCGTGGGCAGCAGCCGGTACAGCAAGAAGTTCAAGCCC
GAGATCGCCATCCGGCCCAAAGTGCGGGACCAAGAGGGCCGGATGAACTACTACTGGACCCTGG
TGGAGCCCGGCGACAAGATCACCTTCGAGGCCACCGGCAATCTGGTGGTGCCCAGATACGCCTTC
GCCATGGAACGGAACGCCGGCAGCGGCATCATCATCAGCGACACCCCCGTGCACGACTGCAACA
CCACCTGTCAGACCCCCAAGGGCGCCATCAACACCAGCCTGCCCTTCCAGAACATCCACCCCATC
ACCATCGGCAAGTGCCCTAAGTACGTGAAGTCCACCAAGCTGCGGCTGGCCACCGGCCTGAGAA
ACGTGCCCAGCATCCAGAGCAGAGGCCTGTTCGGCGCCATTGCCGGCTTTATCGAGGGCGGATGG
ACCGGCATGGTGGACGGGTGGTACGGCTACCACCACCAGAATGAGCAGGGCAGCGGCTACGCCG
CCGACCTGAAGTCCACACAGAACGCCATCGACGAGATCACCAACAAAGTGAACTCCGTGATCGA
GAAGATGAACACCCAGTTCACCGCCGTGGGCAAAGAGTTCAACCACCTGGAAAAGCGGATCGAG
AACCTGAACAAGAAGGTGGACGACGGCTTCCTGGACATCTGGACCTACAACGCCGAGCTGCTGGT
GCTGCTGGAAAACGAGCGGACCCTGGACTACCACGACTCCAACGTGAAGAACCTGTACGAGAAA
GTGCGGAGCCAGCTGAAGAACAACGCCAAAGAGATCGGCAACGGCTGCTTCGAGTTCTACCACA
AGTGCGACAACACCTGTATGGAAAGCGTGAAGAACGGCACCTACGACTACCCCAAGTACAGCGA
GGAAGCCAAGCTGAACCGGGAAGAGATCGACGGCGTGAAGCTGGAAAGCACCCGGATCTACCA
GATCCTGGCCATCTACAGCACCGTGGCCTCCAGCCTGGTGCTGGTGGTGTCCCTGGGCGCCATCTC
CTTTTGGATGTGCTCCAACGGCAGCCTGCAGTGCCGGATCTGCATCTGATGATATCGAATTCAAGG
CCTAGAGCTCCTGGGCCTCATGGGCCTTCCTTTCACTGCC

NotI/EcoRI recognition sites

Start/stop codons

Fig S.2

A/England/195/2009 CLEARFLU version 1 (human codon-optimised)

ATAAGAATGCGGCCGCCACCATGAAGACCATCATTGCCCTGAGCTACATCCTGTGCCTGGTGTTCG
CCCAGAAGATCCCCGGCAACGATAATAGCACCGCCACACTGTGTCTGGGACACCACGCTGTGCCT
AACGGCACCATCGTGAAAACCATCTGCAACGACCGGATCGAAGTGACCAATGCCACCGAGCTGG
TGCAGAATAGCAGCATCGGCGAGATCTGCGACAGCCCTCATCAGATCCTGGACGGCGAGAACTGT
ACCCTGATTGATGCCCTGCTGGGCGACCCTCAGTGTGACGGCTTTCAGAACAAGAAGTGGGACCT
GTTCGTCGAGCGGAGCAAGGCCTACAGCAACTGCTTCCCCTACGATGTGCCTGACTACGCCAGCC
TGAGAAGCCTGGTGGCCTCTTCTGGCACCCTCGAGTTCAACAACGAGAGCTTCAACTGGACCGGC
GTGACCCAGAATGGCACAAGCAGCGCCTGTATCAGACGGTCCAGCAGCAGCTTCTTCAGCAGACT
GAATTGGCTGACCCACCTGAACTACACATACCCCGCTCTGAACGTGACCATGCCTAACAACGAGC
AGTTCGACAAGCTGTACATCTGGGGCGTGCACCATCCTGGCACCGACAAGGATCAGATCTTCCTGT
ATGCCCAGAGCAGCGGCAGAATCACCGTGTCCACCAAGAGAAGCCAGCAGTGCGTGATCCCTTG
CATCGGCAGCAGACCCAGGATCAGAGACATCCCCAGCCGGATCAGCATCTACTGGACAATCGTG
AAGCCCGGCGACATCCTGCTGATCAACAGCACCGGAAACCTGATCGCCCCTCGGGGCTACTTTAA
GATCAGAAGCGGCAAGAGCAGCATCATGCGGAGCGACGCCCCTATCGGCAAGTGCAAGTCTGAG
TGCATCACCCCAAACGGCAGCATCCCCAACGACAAGCCCTTCCAGAACGTGAACAGGATCACCT
ACGGCGCCTGTCCTAGATACGTGAAGCACAACACCCTGAAGCTGGCCACCGGCATGAGAAATGT
GCCCGAGAAGCAGACACAGCAAGGCTTTGGAGCCATTGCCGGCTTCATCGAAAATGGCGCCGAA
GGCATGGTGGATGGCGCCTATGGCTTCAGACACCAGAATAGCGAAGGCAGAGGACAGGCCGCTG
ACCTGAAATCTACACAGGCCGCCATCGACTGCATCAACGGCAAGCTGAACAGACTGATCGGCAA
GACCAACGAGAAGTTCCACCAGATCGAGAAAGAGTTCAGCGAGGTCGAGGGCAGAATCCAGGAC
CTCGAGAAATACGTGGAAGATACCAAGATCGACCTGTGGTCCTACAACGCCGAACTGCTGGTGGC
CCTGGAAAACCAGCACACCATCGATCTGACCGACAGCGAGATGAACAAGCTGTTCGAAAAGACC
AAGAAGCAGCTGCGCGAGAACGCCGAGGATATGGGCAACGGCTGCTTCAAGATCTACCACAAGT
GCGACAACGCCTGCATCGGCTCCATCAGAAACGGCACCTACGACCACAACGTGTACAGAGATGA
GGCCCTGAACAACAGGTTCCAGATCAAAGGCGTGGAACTGAAGTCCGGCTACAAGGATTGGATTC
TGTGGATCAGCTTCGCCATCAGCTGCTTTCTGCTGTGTGTGGCTCTGCTGGGCTTCATCATGTGGCC
TGCCAGAAAGGCAACATCCGGTGCAACATCTGCATCTGATGATATCGAATTCAAGGCCTAG

NotI/EcoRI recognition sites

Start/stop codons

Fig S.3

A/England/195/2009 CLEARFLU version 2 (human codon-optimised)

ATAAGAATGCGGCCGCCACCATGAAGGCTATCCTGGTGGTGCTGCTGTACACCTTCGCCACCGCC
AATGCCGATACACTGTGTATTGGCTACCACGCCAACAACAGCACCGACACCGTGGATACCGTGTG
CGAGAAGAACGTGACCGTGACACACAGCGTGAACATCCTGGAAGATAAGCACAACGGCAAGCTG
TGCAAGCTGAGAGGCGTTGCACCTCTGCACCTGGGCAAGTGTAATATCGCCGGCTGGATCCTGGG
CAACCCTGAGTGTGAAAGCCTGAGCACAGCCAGCAGCTGGTCCTACATCGTGGAAACCAGCAGC
AGCGACAACGGCACCTGTTTTCCCGGCGACTTCATCGACTACGAGGAACTGAGAGAGCAGCTGAG
CAGCGTCAGCAGCTTCGAGAGATTCGAGATCTTCCCCAAGACCTCCAGCTGGCCCAACCACGATT
CTAACAAGGGCGTGACAGCCGCCTGTCCTCATGCCGGCGCTAAGAGCTTCTACAAGAACCTGATC
TGGCTGGTCAAGAAGGGCAACAGCTACCCCAAGCTGAGCAAGAGCTACATCAACGACAAGGGCA
AAGAGGTGCTGGTCCTCTGGGGCATCCACCATCCTTCTACATCTGCCGACCAGCAGAGCCTGTACC
AGAATGCCGATGCCTACGTGTTCGTGGGCAGCAGCAGATACAGCAAGTGCTTCAAGCCCTGTATC
GCCATCAGACCCAAAGTGCGGGACCAAGAGGGCAGAATGAACTACTACTGGACCCTGGTGGAAC
CCGGCGACAAGATCACATTTGAGGCCACCGGCAACCTGGTGGTCCCTAGATATGCCTTCGCCATG
GAAAGAAACGCCGGCAGCGGCATCATCATCAGCGATACACCCGTGCACGACTGCAACACCACCT
GTCAGACACCCAAGGGCGCCATCAATACCAGCCTGCCTTTCCAGAACATTCACCCCATCACCATC
GGCAAGTGCCCCAAATACGTGAAGTCCACAAAGCTGAGACTGGCCACAGGCCTGAGAAACGTGC
CATCCATTCAGAGCCAGCAAGGCTTTGGAGCCGGCGCTGCTTTTATCGAAGGCGGATGGACAGGC
ATGGTGGACGGATGGTACGGCTACCACCATCAGAATGAGCAAGGCAGCGGATACGCCGCCGATC
TGAAGTCTACACAGAACGCCATCGATTGCATCACCAACAAAGTGAACAGCGTGATCGAGAAGATG
AACACCCAGTTCACCGCCGTGGGAAAAGAGTTCAACCACCTGGAAAAGCGCATCGAGAACCTGA
ACAAGAAGGTGGACGACGGCTTCCTGGACATCTGGACCTATAATGCCGAGCTGCTGGTGCTCCTG
GAAAACGAGAGAACCCTGGACTACCACGACAGCAACGTGAAGAACCTGTACGAGAAAGTGCGG
AGCCAGCTGAAGAACAACGCCAAAGAGATCGGCAACGGCTGCTTCGAGTTCTACCACAAGTGCG
ACAATACCTGCATGGAAAGCGTGAAGAATGGCACCTACGACTACCCTAAGTACAGCGAGGAAGC
CAAGCTGAACCGGGAAGAGATTGACGGCGTGAAGCTGGAAAGCACCCGGATCTATCAGATCCTG
GCCATCTACAGCACAGTGGCCTCTAGCCTGGTGCTGGTGGTGTCTCTGGGAGCCATCAGCTTTTGG
ATGTGCAGCAATGGCAGCCTGCAGTGCCGGATCTGCATCTGATGATATCGAATTCAAGGCCTAG

NotI/EcoRI recognition sites

Start/stop codons

Fig S.4

A/Hong Kong/5738/2014 wildtype hemagglutinin (human codon-optimised)

ATAAGAATGCGGCCGCCACCATGAAGACCATCATTGCCCTGAGCTACATCCTGTGCCTGGTGTTCG
CCCAGAAGATCCCCGGCAACGACAACAGCACCGCCACCCTGTGTCTGGGCCACCACGCCGTGCC
CAACGGCACCATCGTGAAAACCATCACCAACGACCGGATCGAAGTGACCAACGCCACCGAGCTG
GTGCAGAACAGCAGCATCGGCGAGATCTGCGACAGCCCCCACCAGATCCTGGACGGCGAGAACT
GCACCCTGATCGACGCCCTGCTGGGCGACCCTCAGTGCGACGGCTTCCAGAACAAGAAATGGGA
CCTGTTCGTGGAAAGAAGCAAGGCCTACAGCAACTGCTACCCCTACGACGTGCCCGACTACGCCA
GCCTGAGAAGCCTGGTGGCCAGCAGCGGCACCCTGGAGTTCAACAACGAGAGCTTCAACTGGAC
CGGCGTGACCCAGAACGGCACCAGCAGCGCCTGCATCAGGCGGAGCAGCAGCAGCTTCTTCAGC
AGACTGAACTGGCTGACCCACCTGAACTACACCTACCCCGCCCTGAACGTGACCATGCCCAACAA
CGAGCAGTTCGACAAGCTGTACATCTGGGGCGTGCACCACCCCGGCACCGATAAGGACCAGATCT
TCCTGTACGCCCAGAGCAGCGGCCGGATCACCGTGTCCACCAAGAGAAGCCAGCAGGCCGTGAT
CCCCAACATCGGCAGCCGGCCCAGAATCCGGGACATCCCCAGCCGGATCAGCATCTACTGGACA
ATCGTGAAGCCCGGCGACATCCTGCTGATCAACTCCACCGGCAACCTGATCGCCCCCAGAGGCTA
CTTCAAGATCAGAAGCGGCAAGAGCAGCATCATGCGGAGCGACGCCCCCATCGGCAAGTGCAAG
AGCGAGTGCATCACCCCCAATGGCAGCATCCCCAACGACAAGCCCTTCCAGAACGTGAACCGGA
TCACCTACGGCGCCTGCCCCAGATACGTGAAGCACAACACCCTGAAGCTGGCCACCGGCATGCG
GAACGTGCCCGAGAAGCAGACCCGGGGCATCTTCGGCGCCATTGCCGGCTTCATCGAGAACGGCT
GGGAGGGCATGGTGGACGGGTGGTACGGCTTCCGGCACCAGAACAGCGAGGGCAGAGGACAGG
CCGCCGACCTGAAGTCTACCCAGGCCGCCATCGACCAGATCAACGGCAAGCTGAACCGGCTGAT
CGGCAAGACCAACGAGAAGTTCCACCAGATCGAGAAAGAATTTTCCGAGGTGGAAGGCCGCATC
CAGGACCTGGAAAAGTACGTGGAAGATACCAAGATCGACCTGTGGTCCTACAACGCCGAGCTGCT
GGTGGCCCTGGAAAACCAGCACACCATCGACCTGACCGACAGCGAGATGAACAAGCTGTTCGAA
AAGACCAAGAAGCAGCTGCGCGAGAACGCCGAGGACATGGGCAACGGCTGCTTTAAGATCTACC
ACAAGTGCGACAACGCCTGCATCGGCAGCATCCGGAACGGCACCTACGACCACAACGTGTACCG
GGACGAGGCCCTGAACAACAGATTCCAGATCAAGGGCGTGGAACTGAAGTCCGGCTACAAGGAC
TGGATTCTGTGGATCAGCTTCGCCATCAGCTGCTTTCTGCTGTGTGTGGCTCTGCTGGGCTTCATCAT
GTGGGCCTGCCAGAAAGGCAACATCCGGTGCAACATCTGCATCTGATGATATCGAATTCAAGGCC
TAGCTGGGCCTCATGGGCCTTCCTTTCACTGCCCGCTTTCCAG

NotI/EcoRI recognition sites

Start/stop codons

Fig S.5

A/Hong Kong/5738/2014 CLEARFLU version 1(human codon-optimised)

ATAAGAATGCGGCCGCCACCATGAAGACCATCATTGCCCTGAGCTACATCCTGTGCCTGGTGTTCG
CCCAGAAGATCCCCGGCAACGATAATAGCACCGCCACACTGTGTCTGGGACACCACGCTGTGCCT
AACGGCACCATCGTGAAAACCATCTGCAACGACCGGATCGAAGTGACCAATGCCACCGAGCTGG
TGCAGAATAGCAGCATCGGCGAGATCTGCGACAGCCCTCATCAGATCCTGGACGGCGAGAACTGT
ACCCTGATTGATGCCCTGCTGGGCGACCCTCAGTGTGACGGCTTTCAGAACAAGAAGTGGGACCT
GTTCGTCGAGCGGAGCAAGGCCTACAGCAACTGCTTCCCCTACGATGTGCCTGACTACGCCAGCC
TGAGAAGCCTGGTGGCCTCTTCTGGCACCCTCGAGTTCAACAACGAGAGCTTCAACTGGACCGGC
GTGACCCAGAATGGCACAAGCAGCGCCTGTATCAGACGGTCCAGCAGCAGCTTCTTCAGCAGACT
GAATTGGCTGACCCACCTGAACTACACATACCCCGCTCTGAACGTGACCATGCCTAACAACGAGC
AGTTCGACAAGCTGTACATCTGGGGCGTGCACCATCCTGGCACCGACAAGGATCAGATCTTCCTGT
ATGCCCAGAGCAGCGGCAGAATCACCGTGTCCACCAAGAGAAGCCAGCAGTGCGTGATCCCTTG
CATCGGCAGCAGACCCAGGATCAGAGACATCCCCAGCCGGATCAGCATCTACTGGACAATCGTG
AAGCCCGGCGACATCCTGCTGATCAACAGCACCGGAAACCTGATCGCCCCTCGGGGCTACTTTAA
GATCAGAAGCGGCAAGAGCAGCATCATGCGGAGCGACGCCCCTATCGGCAAGTGCAAGTCTGAG
TGCATCACCCCAAACGGCAGCATCCCCAACGACAAGCCCTTCCAGAACGTGAACAGGATCACCT
ACGGCGCCTGTCCTAGATACGTGAAGCACAACACCCTGAAGCTGGCCACCGGCATGAGAAATGT
GCCCGAGAAGCAGACACAGCAAGGCTTTGGAGCCATTGCCGGCTTCATCGAAAATGGCGCCGAA
GGCATGGTGGATGGCGCCTATGGCTTCAGACACCAGAATAGCGAAGGCAGAGGACAGGCCGCTG
ACCTGAAATCTACACAGGCCGCCATCGACTGCATCAACGGCAAGCTGAACAGACTGATCGGCAA
GACCAACGAGAAGTTCCACCAGATCGAGAAAGAGTTCAGCGAGGTCGAGGGCAGAATCCAGGAC
CTCGAGAAATACGTGGAAGATACCAAGATCGACCTGTGGTCCTACAACGCCGAACTGCTGGTGGC
CCTGGAAAACCAGCACACCATCGATCTGACCGACAGCGAGATGAACAAGCTGTTCGAAAAGACC
AAGAAGCAGCTGCGCGAGAACGCCGAGGATATGGGCAACGGCTGCTTCAAGATCTACCACAAGT
GCGACAACGCCTGCATCGGCTCCATCAGAAACGGCACCTACGACCACAACGTGTACAGAGATGA
GGCCCTGAACAACAGGTTCCAGATCAAAGGCGTGGAACTGAAGTCCGGCTACAAGGATTGGATTC
TGTGGATCAGCTTCGCCATCAGCTGCTTTCTGCTGTGTGTGGCTCTGCTGGGCTTCATCATGTGGGCC
TGCCAGAAAGGCAACATCCGGTGCAACATCTGCATCTGATGATATCGAATTCAAGGCCTAG

NotI/EcoRI recognition sites

Start/stop codons

Fig S.6

A/Hong Kong/5738/2014 CLEARFLU version 2 (human codon-optimised)

ATAAGAATGCGGCCGCCACCATGAAGACCATCATTGCCCTGAGCTACATCCTGTGCCTGGTGTTCG
CCCAGAAGATCCCCGGCAACGATAATAGCACCGCCACACTGTGTCTGGGACACCACGCTGTGCCT
AACGGCACCATCGTGAAAACCATCTGCAACGACCGGATCGAAGTGACCAATGCCACCGAGCTGG
TGCAGAATAGCAGCATCGGCGAGATCTGCGACAGCCCTCATCAGATCCTGGACGGCGAGAACTGT
ACCCTGATTGATGCCCTGCTGGGCGACCCTCAGTGTGACGGCTTTCAGAACAAGAAGTGGGACCT
GTTCGTCGAGCGGAGCAAGGCCTACAGCAACTGCTTCCCCTACGATGTGCCTGACTACGCCAGCC
TGAGAAGCCTGGTGGCCTCTTCTGGCACCCTCGAGTTCAACAACGAGAGCTTCAATTGGACCGGC
GTGACCCAGAATGGCACCTCTAGCGCCTGTATCAGACGGTCCAGCAGCAGCTTCTTCAGCAGACT
GAATTGGCTGACCCACCTGAACTACACATACCCCGCTCTGAACGTGACCATGCCTAACAACGAGC
AGTTCGACAAGCTGTACATCTGGGGCGTGCACCATCCTGGCACCGACAAGGATCAGATCTTCCTGT
ATGCCCAGAGCAGCGGCAGAATCACCGTGTCCACCAAGAGAAGCCAGCAGTGCGTGATCCCTTG
CATCGGCAGCAGACCCAGGATCAGAGACATCCCCAGCCGGATCAGCATCTACTGGACAATCGTG
AAGCCCGGCGACATCCTGCTGATCAACAGCACCGGAAACCTGATCGCCCCTCGGGGCTACTTTAA
GATCAGAAGCGGCAAGAGCAGCATCATGCGGAGCGACGCCCCTATCGGCAAGTGCAAGTCTGAG
TGCATCACCCCAAACGGCAGCATCCCCAACGACAAGCCCTTCCAGAACGTGAACAGGATCACCT
ACGGCGCCTGTCCTAGATACGTGAAGCACAACACCCTGAAGCTGGCCACCGGCATGAGAAATGT
GCCCGAGAAGCAGACACAGCAAGGCTTTGGAGCCGGCGCTGCCTTTATCGAGAATGGCTGGGAA
GGCATGGTGGACGGATGGTACGGCTTCAGACACCAGAATAGCGAAGGCAGAGGACAGGCCGCTG
ACCTGAAATCTACACAGGCCGCCATCGACTGCATCAACGGCAAGCTGAACAGACTGATCGGCAA
GACCAACGAGAAGTTCCACCAGATCGAGAAAGAGTTCAGCGAGGTCGAGGGCAGAATCCAGGAC
CTCGAGAAATACGTGGAAGATACCAAGATCGACCTGTGGTCCTACAACGCCGAACTGCTGGTGGC
CCTGGAAAACCAGCACACCATCGATCTGACCGACAGCGAGATGAACAAGCTGTTCGAAAAGACC
AAGAAGCAGCTGCGCGAGAACGCCGAGGATATGGGCAATGGCTGCTTCAAGATCTACCACAAGT
GCGACAACGCCTGCATCGGCTCCATCAGAAACGGCACCTACGACCACAACGTGTACAGAGATGA
GGCCCTGAACAACCGGTTCCAGATCAAAGGCGTGGAACTGAAGTCCGGCTACAAGGACTGGATC
CTGTGGATCAGCTTCGCCATCAGCTGCTTTCTGCTGTGTGTGGCTCTGCTGGGCTTCATCATGTGGGC
CTGCCAGAAAGGCAACATCCGGTGCAACATCTGCATCTGATGATATCGAATTCAAGGCCTAG

NotI/EcoRI recognition sites

Start/stop codons

Fig S.7

A/Hong Kong/125/2017 wildtype hemagglutinin (human codon-optimised)

ATAAGAATGCGGCCGCCACCATGAACACCCAGATCCTGGTGTTTGCCCTGATCGCCATCATTCCCA
CCAACGCCGATAAGATCTGTCTGGGCCACCACGCCGTGTCCAACGGCACAAAAGTGAACACACT
GACCGAGCGCGGCGTGGAAGTGGTCAATGCCACAGAGACAGTGGAACGGACAAACATCCCCAG
AATCTGCAGCAAGGGCAAGAGAACCGTGGATCTGGGACAGTGTGGCCTGCTGGGAACAATTACA
GGCCCTCCTCAGTGCGACCAGTTCCTGGAATTTTCCGCCGACCTGATCATCGAGCGGAGAGAAGG
CTCCGATGTGTGCTACCCCGGCAAGTTCGTGAACGAGGAAGCCCTGAGACAGATCCTGAGAGAGA
GCGGCGGCATCGACAAAGAAACCATGGGCTTCACCTACAACGGCATCAGGACCAATGGCGTGAC
CAGCGCCTGTAAAAGAAGCGGCAGCAGCTTCTACGCCGAGATGAAGTGGCTGCTGAGCAACACC
GACAACGCCGCCTTTCCACAGATGACCAAGAGCTACAAGAACACCCGCAAGAGCCCCGCCATCA
TCGTGTGGGGAATCCACCATTCTGTGTCCACCGCCGAGCAGACAAAGCTGTACGGCTCTGGCAAC
AAGCTGGTCACCGTGGGCAGCAGCAATTACCAGCAGAGCTTTGTGCCAAGTCCAGGCGCCAGACC
TCAAGTGAATGGCCTGAGCGGCAGAATCGACTTCCACTGGCTGATTCTGAACCCCAACGACACCG
TGACCTTCAGCTTCAACGGCGCCTTTATCGCCCCTGACAGAGCCAGCTTTCTGAGAGGCAAGAGCA
TGGGCATCCAGTCTGGCGTGCAAGTGGATGCCAATTGCGAGGGCGATTGCTACCACTCTGGCGGC
ACCATCATCAGCAACCTGCCTTTCCAGAACATCGACAGCAGAGCCGTGGGCAAGTGCCCCAGATA
CGTGAAACAAAGAAGCCTGCTGCTGGCCACCGGCATGAAGAATGTGCCTGAGATCCCTAAAGGC
AGAGGCCTGTTTGGCGCCATTGCCGGCTTTATCGAGAATGGCTGGGAGGGACTGATCGACGGATG
GTACGGCTTCAGACACCAGAATGCCCAAGGCGAGGGAACAGCCGCCGATTACAAAAGCACACA
GAGCGCCATCGACCAGATCACCGGCAAGCTGAACAGACTGATTGCCAAGACCAACCAGCAGTTC
GAGCTGATCGACAACGAGTTCAACGAGGTGGAAAAGCAGATCGGCAACGTGATCAACTGGACCC
GGGACAGCATCACCGAAGTGTGGTCTTACAACGCCGAGCTGCTGATCGCTATGGAAAACCAGCAC
ACCATCGACCTGGCCGACAGCGAGATGGACAAGCTGTATGAGAGAGTGAAGAGACAGCTGCGCG
AGAACGCCGAGGAAGATGGCACAGGCTGCTTCGAGATCTTCCACAAGTGCGACGACGACTGCAT
GGCCAGCATCCGGAACAACACCTACGACCACCGGAAGTACCGGGAAGAAGCCATGCAGAACAG
AATCCAGATCGACCCCGTGAAGCTGAGCAGCGGCTACAAGGATGTGATCCTGTGGTTTAGCTTCGG
CGCCAGCTGCTTCATCCTGCTGGCTATTGTGATGGGCCTCGTGTTCATCTGCGTGAAGAACGGCAAC
ATGCGGTGCACCATCTGCATCTGATGATATCGAATTCAAGGCCTAGGAGCTCGGAGCACAAGACT
GGCCTCATGGCCTTCCTTTCACTGCC

NotI/EcoRI recognition sites

Start/stop codons

Fig S.8

A/Hong Kong/125/2017 CLEARFLU version 2 (human codon-optimised)

ATAAGAATGCGGCCGCCACCATGAACACCCAGATCCTGGTGTTTGCCCTGATCGCCATCATTCCCA
CCAACGCCGATAAGATCTGTCTGGGCCACCACGCCGTGTCCAACGGCACAAAAGTGAACACACT
GTGCGAGCGCGGCGTGGAAGTGGTCAATGCCACAGAGACAGTGGAACGGACAAACATCCCCAGA
ATCTGCAGCAAGGGCAAGAGAACCGTGGATCTGGGACAGTGTGGCCTGCTGGGAACAATTACAG
GCCCTCCTCAGTGCGACCAGTTCCTGGAATTTTCCGCCGACCTGATCATCGAGCGGAGAGAAGGC
AGCGACGTGTGCTTCCCTGGCAAGTTCGTGAACGAGGAAGCCCTGAGACAGATCCTGAGAGAGAG
CGGCGGCATCGACAAAGAAACCATGGGCTTCACCTACAACGGCATCAGGACCAATGGCGTGACC
AGCGCCTGTAAAAGAAGCGGCAGCAGCTTCTACGCCGAGATGAAGTGGCTGCTGAGCAACACCG
ACAACGCCGCCTTTCCACAGATGACCAAGAGCTACAAGAACACCCGCAAGAGCCCCGCCATCAT
CGTGTGGGGAATCCACCATTCTGTGTCCACCGCCGAGCAGACAAAGCTGTACGGCTCTGGCAACA
AGCTGGTCACCGTGGGCAGCAGCAATTACCAGCAGTGCTTTGTGCCCTGTCCTGGCGCTAGACCTC
AAGTGAATGGCCTGAGCGGCAGAATCGACTTCCACTGGCTGATTCTGAACCCCAACGACACCGTG
ACCTTCAGCTTCAACGGCGCCTTTATCGCCCCTGACAGAGCCAGCTTTCTGAGAGGCAAGAGCATG
GGCATCCAGTCTGGCGTGCAAGTGGATGCCAATTGCGAGGGCGATTGCTACCACTCTGGCGGCAC
CATCATCAGCAACCTGCCTTTCCAGAACATCGACAGCAGAGCCGTGGGCAAGTGCCCCAGATACG
TGAAACAAAGAAGCCTGCTGCTGGCCACCGGCATGAAGAATGTGCCTGAGATCCCTAAGGGCCA
GCAAGGATTTGGAGCCGGCGCAGCCTTTATTGAGAATGGCTGGGAGGGACTGATCGACGGATGGT
ACGGCTTCAGACACCAGAATGCCCAAGGCGAGGGAACAGCCGCCGATTACAAAAGCACACAGA
GCGCCATCGACTGCATCACCGGCAAGCTGAACAGACTGATTGCCAAGACCAACCAGCAGTTCGA
GCTGATCGACAACGAGTTCAACGAGGTGGAAAAGCAGATCGGCAACGTGATCAACTGGACCCGG
GACAGCATCACCGAAGTGTGGTCTTACAACGCCGAGCTGCTGATCGCTATGGAAAACCAGCACAC
CATCGACCTGGCCGACAGCGAGATGGACAAGCTGTATGAGAGAGTGAAGAGACAGCTGCGCGAG
AACGCCGAGGAAGATGGCACAGGCTGCTTCGAGATCTTCCACAAGTGCGACGACGACTGCATGG
CCAGCATCCGGAACAACACCTACGACCACCGGAAGTACCGGGAAGAAGCCATGCAGAACAGAA
TCCAGATCGACCCCGTGAAGCTGAGCAGCGGCTACAAGGATGTGATCCTGTGGTTCAGCTTCGGC
GCCAGCTGTTTCATCCTGCTGGCTATCGTGATGGGCCTCGTGTTCATCTGCGTGAAGAACGGCAAC
ATGCGGTGCACCATCTGCATCTGATGATATCGAATTCAAGGCCTAG

NotI/EcoRI recognition sites

Start/stop codons

Fig S.9

A/Hong Kong/125/2017 CLEARFLU version 3 (human codon-optimised)

ATAAGAATGCGGCCGCCACCATGAACACCCAGATCCTGGTGTTTGCCCTGATCGCCATCATTCCCA
CCAACGCCGATAAGATCTGTCTGGGCCACCACGCCGTGTCCAACGGCACAAAAGTGAACACACT
GTGCGAGCGCGGCGTGGAAGTGGTCAATGCCACAGAGACAGTGGAACGGACCAACATCCCCCGG
ATCTGCAGCAAGGGCAAGAGAACAGTGGATCTGGGCCAGTGTGGCCTGCTGGGAACAATTACAG
GCCCTCCTCAGTGCGACCAGTTCCTGGAATTTTCCGCCGACCTGATCATCGAGCGGAGAGAAGGCT
CCGATGTGTGCTACCCCGGCAAGTTCGTGAACGAGGAAGCCCTGAGACAGATCCTGAGAGAGAG
CGGCGGCATCGACAAAGAAACCATGGGCTTCACCTACAACGGCATCAGAACAAATGGCGTGACC
AGCGCCTGTAAGAGAAGCGGCAGCAGCTTCTACGCCGAGATGAAGTGGCTGCTGAGCAACACCG
ACAACGCCGCCTTTCCACAGATGACCAAGAGCTACAAGAACACCCGCAAGAGCCCTGCCATCAT
CGTGTGGGGAATCCACCACTCTGTGTCTACCGCCGAGCAGACAAAGCTGTACGGCTCTGGCAACA
AGCTGGTCACCGTGGGCAGCAGCAATTACCAGCAGTGCTTTGTGCCCTGTCCTGGCGCTAGACCTC
AAGTGAATGGCCTGAGCGGCAGAATCGACTTCCACTGGCTGATTCTGAACCCCAACGACACCGTG
ACCTTCAGCTTCAACGGCGCCTTTATCGCCCCTGACAGAGCCAGCTTTCTGAGAGGCAAGAGCATG
GGCATCCAGTCTGGCGTGCAAGTGGATGCCAATTGCGAGGGCGATTGCTACCACTCTGGCGGCAC
CATCATCAGCAACCTGCCTTTCCAGAACATCGACAGCCGGGCCGTGGGCAAGTGCCCCAGATACG
TGAAACAGAGAAGCCTGCTGCTGGCCACCGGCATGAAGAATGTGCCTGAGATCCCTAAGGGCCA
GCAAGGATTTGGAGCCGGCGCTGCCTTTATTGAGAATGGCTGGGAGGGACTGATCGACGGATGGT
ACGGCTTCAGACACCAGAATGCCCAAGGCGAGGGAACAGCCGCCGATTACAAAAGCACACAGA
GCGCCATCGACTGCATCACCGGCAAGCTGAACAGACTGATTGCCAAGACCAACCAGCAGCCTGA
GCTGATCGACAACGAGCCCAACGAGGTGGAAAAGCAGATCGGCAACGTGATCAACTGGACCCGG
GACAGCATCACCGAAGTGTGGTCTTACAATGCCGAGCTGCTGATCGCCATGGAAAACCAGCACAC
CATCGATCTGGCCGACAGCGAGATGGACAAGCTGTATGAGAGAGTGAAGAGACAGCTGCGCGAG
AACGCCGAGGAAGATGGCACAGGCTGCTTCGAGATCTTCCACAAGTGCGACGACGACTGCATGG
CCAGCATCCGGAACAACACCTACGACCACCGGAAGTACCGGGAAGAAGCCATGCAGAACAGAA
TCCAGATCGACCCCGTGAAGCTGAGCAGCGGCTACAAGGATGTGATCCTGTGGTTCAGCTTCGGC
GCCAGCTGTTTCATCCTGCTGGCTATCGTGATGGGCCTCGTGTTCATCTGCGTGAAGAACGGCAAC
ATGCGGTGCACCATCTGCATCTGATGATATCGAATTCAAGGCCTAG

Fig S.10

Truncated A/Hong Kong/125/2017 CLEARFLU version 3 segment in two viral clones

AGCAAAAGCAGGGGAAAATAAAAACAACCAAATAGAAGGCAAACCTACTGGTCCTGTTACGTGC
ACTTGCAGCTGCAGTAGCGCGGCCGCCACC- 1620nt deletion including original start codon-
GTGATGGGCCTCGTGTTCATCTGCGTGAAGAACGGCAACATGCGGTGCACCATCTGCATCTGATGA
TATCGAATTCGGAAAGTGTAAGAAATGGGACTTATGATTATCCCAAATATTCAGAAGAGTCAAAGT
TGAACAGGGAAAAGGTAGATGGAGTGAAATTGGAATCAATGGGGATCTATCAGATTCTGGCGATC
TACTCAACTGTCGCCAGTTCACTGGTGCTTTTGGTCTCCCTGGGGGCAATCAGTTTCTGGATGTGTTC
TAATGGATCTTTGCAGTGCAGAATATGCATCTGAGATTAGAATTTCAGAAATATGAGGAAAAACAC
CCTTGTTTCTACT

Uni12/13 primers

NotI/EcoRI recognition sites

Start/stop codons

CLEARFLU sequence

Fig S.11

Alignment of full length and truncated A/Hong Kong/125/2017 CLEARFLU version 3 viral segments found in round 6 clones

```
1   AGCAAAAGCAGGGGAAAATAAAAACAACCAAATAGAAGGCAAACCTACTGGTCCTGTTAC 60
    ||||||||||||||||||||||||||||||||||||||||||||||||||||||||||||
1   AGCAAAAGCAGGGGAAAATAAAAACAACCAAATAGAAGGCAAACCTACTGGTCCTGTTAC 60

                                     start
61  GTGCACTTGCAGCTGCAGTAGCGCGGCCGCCACCATGAACACCCAGATCCTGGTGTTTGC 120
    ||||||||||||||||||||||||||||||||||
61  GTGCACTTGCAGCTGCAGTAGCGCGGCCGCCACC-------------------------- 94

121 CCTGATCGCCATCATTCCCACCAACGCCGATAAGATCTGTCTGGGCCACCACGCCGTGTC 180
94  ------------------------------------------------------------ 94

181 CAACGGCACAAAAGTGAACACACTGTGCGAGCGCGGCGTGGAAGTGGTCAATGCCACAGA 240
94  ------------------------------------------------------------ 94

241 GACAGTGGAACGGACCAACATCCCCCGGATCTGCAGCAAGGGCAAGAGAACAGTGGATCT 300
94  ------------------------------------------------------------ 94

301 GGGCCAGTGTGGCCTGCTGGGAACAATTACAGGCCCTCCTCAGTGCGACCAGTTCCTGGA 360
94  ------------------------------------------------------------ 94

361 ATTTTCCGCCGACCTGATCATCGAGCGGAGAGAAGGCTCCGATGTGTGCTACCCCGGCAA 420
94  ------------------------------------------------------------ 94

421 GTTCGTGAACGAGGAAGCCCTGAGACAGATCCTGAGAGAGAGCGGCGGCATCGACAAAGA 480
94  ------------------------------------------------------------ 94

481 AACCATGGGCTTCACCTACAACGGCATCAGAACAAATGGCGTGACCAGCGCCTGTAAGAG 540
94  ------------------------------------------------------------ 94

541 AAGCGGCAGCAGCTTCTACGCCGAGATGAAGTGGCTGCTGAGCAACACCGACAACGCCGC 600
94  ------------------------------------------------------------ 94

601 CTTTCCACAGATGACCAAGAGCTACAAGAACACCCGCAAGAGCCCTGCCATCATCGTGTG 660
94  ------------------------------------------------------------ 94
```

661 GGGAATCCACCACTCTGTGTCTACCGCCGAGCAGACAAAGCTGTACGGCTCTGGCAACAA 720

94 -- 94

721 GCTGGTCACCGTGGGCAGCAGCAATTACCAGCAGTGCTTTGTGCCCTGTCCTGGCGCTAG 780

94 -- 94

781 ACCTCAAGTGAATGGCCTGAGCGGCAGAATCGACTTCCACTGGCTGATTCTGAACCCCAA 840

94 -- 94

841 CGACACCGTGACCTTCAGCTTCAACGGCGCCTTTATCGCCCCTGACAGAGCCAGCTTTCT 900

94 -- 94

901 GAGAGGCAAGAGCATGGGCATCCAGTCTGGCGTGCAAGTGGATGCCAATTGCGAGGGCGA 960

94 -- 94

961 TTGCTACCACTCTGGCGGCACCATCATCAGCAACCTGCCTTTCCAGAACATCGACAGCCG 1020

94 -- 94

1021 GGCCGTGGGCAAGTGCCCNAGATACGTGAAACAGAGAAGCCTGCTGCTGGCCACCGGCAT 1080

94 -- 94

1081 GAAGAATGTGCCTGAGATCCCTAAGGGCCAGCAAGGATTTGGAGCCGGCGCTGCCTTTAT 1140

94 -- 94

1141 TGAGAATGGCTGGGAGGGACTGATCGACGGATGGTACGGCTTCAGACACCAGAATGCCCA 1200

94 -- 94

1201 AGGCGAGGGAACAGCCGCCGATTACAAAAGCACACAGAGCGCCATCGACTGCATCACCGG 1260

94 -- 94

1261 CAAGCTGAACAGACTGATTGCCAAGACCAACCAGCAGCCTGAGCTGATCGACAACGAGCC 1320

94 -- 94

1321 CAACGAGGTGGAAAAGCAGATCGGCAACGTGATCAACTGGACCCGGGACAGCATCACCGA 1380

94 -- 94

```
1381 AGTGTGGTCTTACAATGCCGAGCTGCTGATCGCCATGGAAAACCAGCACACCATCGATCT 1440

94   ------------------------------------------------------------ 94

1441 GGCCGACAGCGAGATGGACAAGCTGTATGAGAGAGTGAAGAGACAGCTGCGCGAGAACGC 1500

94   ------------------------------------------------------------ 94

1501 CGAGGAAGATGGCACAGGCTGCTTCGAGATCTTCCACAAGTGCGACGACGACTGCATGGC 1560

94   ------------------------------------------------------------ 94

1561 CAGCATCCGGAACAACACCTACGACCACCGGAAGTACCGGGAAGAAGCCATGCAGAACAG 1620

94   ------------------------------------------------------------ 94

1621 AATCCAGATCGACCCCGTGAAGCTGAGCAGCGGCTACAAGGATGTGATCCTGTGGTTCAG 1680

94   ------------------------------------------------------------ 94

                                        new start
1681 CTTCGGCGCCAGCTGTTTCATCCTGCTGGCTATCGTGATGGGCCTCGTGTTCATCTGCGT 1740
                                       ||||||||||||||||||||||||||
94   ----------------------------------GTGATGGGCCTCGTGTTCATCTGCGT 120

1741 GAAGAACGGCAACATGCGGTGCACCATCTGCATCTGATGATATCGAATTCGGAAAGTGTA 1800
     ||||||||||||||||||||||||||||||||||||||||||||||||||||||||||||
121  GAAGAACGGCAACATGCGGTGCACCATCTGCATCTGATGATATCGAATTCGGAAAGTGTA 180

1801 AGAAATGGGACTTATGATTATCCCAAATATTCAGAAGAGTCAAAGTTGAACAGGGAAAAG 1860
     ||||||||||||||||||||||||||||||||||||||||||||||||||||||||||||
181  AGAAATGGGACTTATGATTATCCCAAATATTCAGAAGAGTCAAAGTTGAACAGGGAAAAG 240

1861 GTAGATGGAGTGAAATTGGAATCAATGGGGATCTATCAGATTCTGGCGATCTACTCAACT 1920
     ||||||||||||||||||||||||||||||||||||||||||||||||||||||||||||
241  GTAGATGGAGTGAAATTGGAATCAATGGGGATCTATCAGATTCTGGCGATCTACTCAACT 300

1921 GTCGCCAGTTCACTGGTGCTTTTGGTCTCCCTGGGGGCAATCAGTTTCTGGATGTGTTCT 1980
     ||||||||||||||||||||||||||||||||||||||||||||||||||||||||||||
301  GTCGCCAGTTCACTGGTGCTTTTGGTCTCCCTGGGGGCAATCAGTTTCTGGATGTGTTCT 360

1981 AATGGATCTTTGCAGTGCAGAATATGCATCTGAGATTAGAATTTCAGAAATATGAGGAAA 2040
     ||||||||||||||||||||||||||||||||||||||||||||||||||||||||||||
361  AATGGATCTTTGCAGTGCAGAATATGCATCTGAGATTAGAATTTCAGAAATATGAGGAAA 420

2041 AACACCCTTGTTTCTACT 2058
     ||||||||||||||||||
421  AACACCCTTGTTTCTACT 438
```

Fig S.12

Neutralisation of T148A S-Flu viruses by neuraminidase inhibiting antibody AG7C and chemical inhibitor oseltamivir

Antibodies were diluted to 20µg/ml and oseltamivir to 200nM before being added to virus in doubling dilutions. 4A14 and 3CSY were used as control antibodies as they neutralise S-Flu viruses coated in H7 (A/Hong Kong/125/2017) hemagglutinin and Zaire-GP respectively. Cell entry was measured by eGFP fluorescence. Points and error bars indicate the mean and standard deviation of the two duplicate measures. Data are shown from a single experiment which was repeated with the same result.

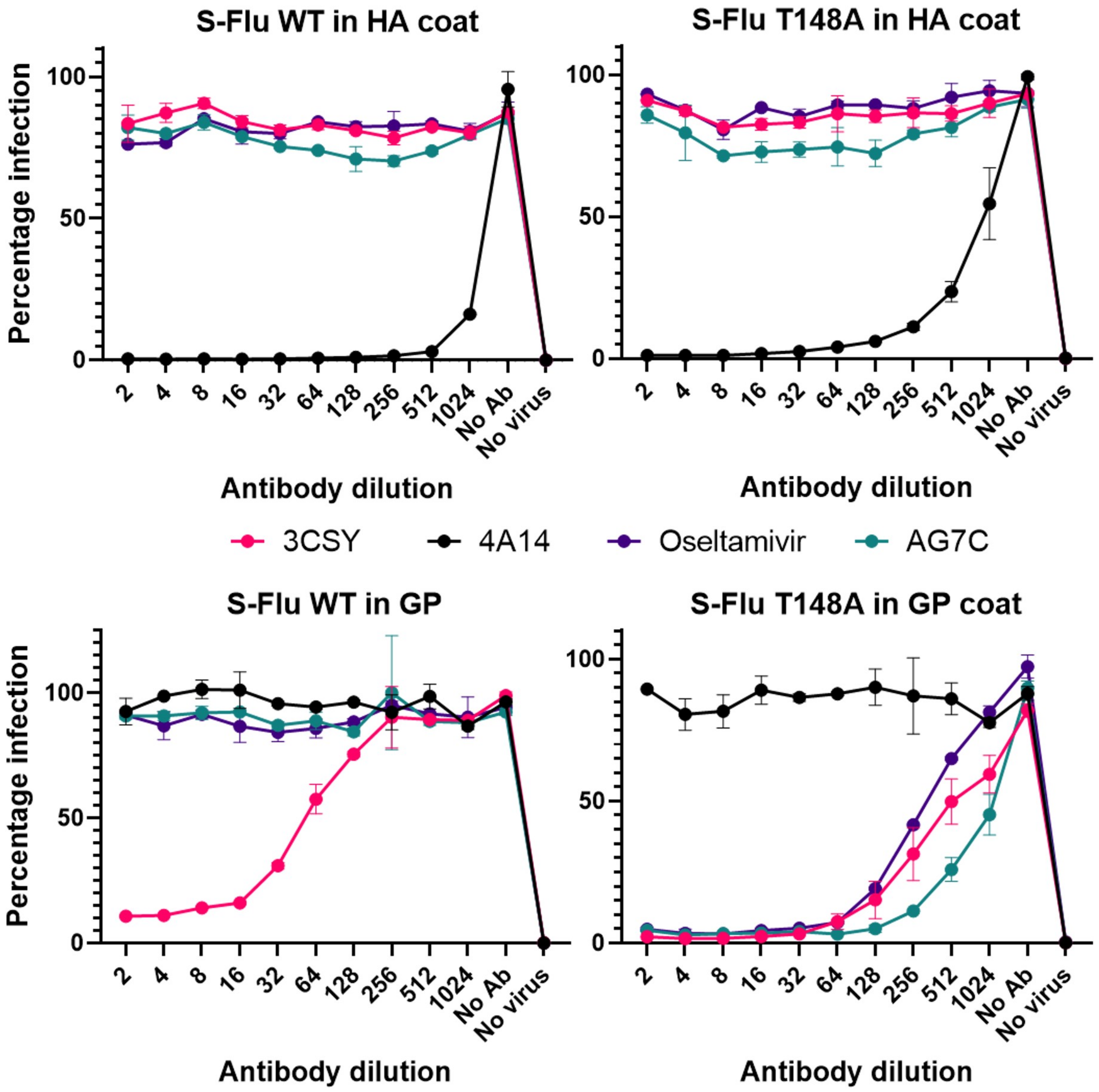

Fig S.13

Neuraminidase mediated agglutination of RBCs by T148A S-Flus

Viruses were titrated in doubling dilutions and incubated with 5µl of 200nM oseltamivir and/or 2µl of antibody 4A14 at 25µg/ml for 1 hour before adding RBCs. Boxes indicate wells positive for agglutination. Dashed boxes indicate partial positives in which a red pellet is present but does not form a teardrop and the well has a pinkish hue. All assays were repeated with the same results.
A. S-Flus were titrated in doubling dilutions in duplicate in the absence of inhibitors.
B,C&D. S-Flus which agglutinated RBCs in part A were titrated in the presence of neuraminidase inhibitor oseltamivir and/or H7 antibody 4A14.

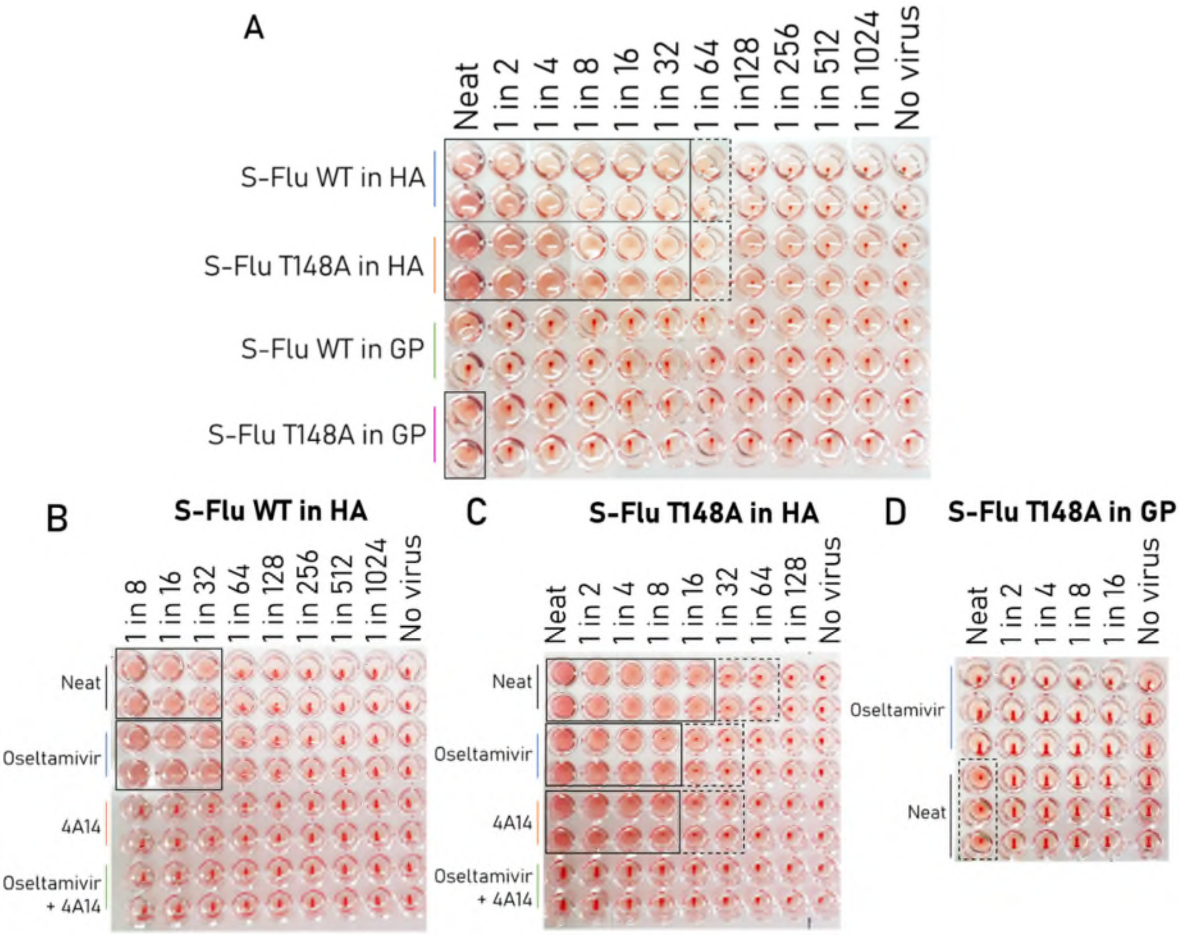

Supplement: Supplemental figures — Figures S1 to S13. [file jvi.00331-24-s0001.pdf]
